# A Complex Network Approach to Distributional Semantic Models

**DOI:** 10.1371/journal.pone.0136277

**Published:** 2015-08-21

**Authors:** Akira Utsumi

**Affiliations:** Department of Informatics, The University of Electro-Communications, Tokyo, Japan; Hangzhou Normal University, CHINA

## Abstract

A number of studies on network analysis have focused on language networks based on free word association, which reflects human lexical knowledge, and have demonstrated the small-world and scale-free properties in the word association network. Nevertheless, there have been very few attempts at applying network analysis to distributional semantic models, despite the fact that these models have been studied extensively as computational or cognitive models of human lexical knowledge. In this paper, we analyze three network properties, namely, small-world, scale-free, and hierarchical properties, of semantic networks created by distributional semantic models. We demonstrate that the created networks generally exhibit the same properties as word association networks. In particular, we show that the distribution of the number of connections in these networks follows the truncated power law, which is also observed in an association network. This indicates that distributional semantic models can provide a plausible model of lexical knowledge. Additionally, the observed differences in the network properties of various implementations of distributional semantic models are consistently explained or predicted by considering the intrinsic semantic features of a word-context matrix and the functions of matrix weighting and smoothing. Furthermore, to simulate a semantic network with the observed network properties, we propose a new growing network model based on the model of Steyvers and Tenenbaum. The idea underlying the proposed model is that both preferential and random attachments are required to reflect different types of semantic relations in network growth process. We demonstrate that this model provides a better explanation of network behaviors generated by distributional semantic models.

## Introduction

How word meaning is represented in human memory is a longstanding problem that has attracted the interest of linguists, philosophers, psychologists and other scholars. A number of theories and models for lexical representation or the mental lexicon—e.g., semantic networks [1, 2], feature-list theory [3, 4], and prototype theory [5, 6]—have been proposed, and their validity has been examined and debated using psychological experiments [7, 8]. Computational modeling, especially the connectionist model [9], has also been employed to explore lexical representation. Brain science has revealed the biological and neuroanatomical basis of the mental lexicon [10].

Recently, network science or network analysis has emerged as a new research methodology for the study of language in general and semantic representation in particular [11–13]. Network analysis takes as input a network produced from the observable data (i.e., word cooccurrence and word association) about the mental lexicon or human semantic memory, and reveals the properties of the network. These network properties provide information about the structure of the mental lexicon and the cognitive mechanism underlying the semantic structure, neither of which is directly observable from the data [12]. Furthermore, despite their simplicity, network models that simulate the observed network properties can provide valuable insight into the process of lexical development by which these network properties emerge. Therefore, a large number of network studies have investigated semantic or lexical representation and its related phenomena such as word co-occurrence [14–17], the phonological lexicon [18, 19], thesaurus [20, 21], and verbal fluency [22]. The most studied phenomenon among these is free word association [23, 24], because it reflects human lexical knowledge acquired through world experience, thereby revealing the structure of human semantic memory or the mental lexicon more directly and efficiently than other lexical phenomena.

Network studies on word association have demonstrated the small-world and scale-free properties of semantic networks [21, 25–29]. For an association network where each word is represented by a node and an association relation between two words is represented by an edge joining the corresponding nodes, the small-world property indicates that any two word nodes are connected by traversing only a few edges, even if the network is highly clustered locally. The scale-free property indicates that most word nodes are poorly connected, while a relatively small number of words are highly connected; as a result, the distribution of the number of connections for each node follows a power law. All existing studies agree on the small-world property of the association network, but some studies [29] suggest that the association network is not completely scale-free; rather the network is characterized by a power law truncated by an exponential cutoff, where the most connected words have a smaller connection than would be expected in a purely power-law distributed network. These network properties are expected to reveal the cognitive mechanism underlying the structure of the mental lexicon [12]. For example, the small-world structure sheds light on an efficient search process in semantic memory. Investigating various network models that generate scale-free networks provides valuable insight about psychological processes involved in lexical development [21].

Another emerging topic in the study of lexical representation is the distributional semantic model (DSM) [30–32] (also known as a vector space model). Since Landauer et al. [33] published their seminal study on latent semantic analysis (LSA), the DSM framework has been extensively studied as a computational or cognitive model of human semantic memory. In DSMs, the lexical meaning of a word is represented by a high-dimensional vector in a semantic space, and the degree of semantic relatedness between any two words can easily be computed from their vectors. Word vectors are constructed from a corpus by observing distributional statistics of word occurrence. DSMs have been demonstrated to explain a number of cognitive phenomena relevant to semantic knowledge or the mental lexicon such as similarity judgment [33, 34], semantic priming [35], visual attention to semantically relevant objects [36], and embodiment [37, 38]. The primary advantage of DSMs is that, despite their simplicity, they are very useful for a variety of applications in not only cognitive modeling but also natural language processing; hence, an increasing number of studies have focused on DSMs and proposed a variety of new methods for constructing word vectors.

However, these two emerging topics have not yet been linked in the literature, and DSMs have rarely been investigated in network analysis, in contrast to the growing network-analytic interest in word association and the lexical structure of language. It is quite important in the study of DSMs to examine whether they exhibit the same network properties as observed in the analysis of word association and other language networks. In so doing, network analysis can provide evidence for (or against) the validity of DSMs as a cognitive model of semantic memory or the mental lexicon.

One notable exception is the study by Steyvers and Tenenbaum [21], which investigated whether LSA [31, 33], one of the most popular versions of DSMs, exhibits the same network structure as word association. They found that LSA networks were small-world, but not scale-free. A subsequent study by Griffiths et al. [39] obtained the same result; the scale-free property of word association is difficult to reproduce in LSA, although it is appropriately simulated by the topic model based on latent Dirichlet allocation. From these results, they concluded that LSA is limited as a model of human semantic memory. However, their findings do not imply that DSMs generally fail to model semantic memory, because a variety of methods for constructing semantic spaces other than LSA are devised in the DSM framework [30, 32]. Furthermore, as Morais et al. [29] pointed out, their analysis of the scale-free property was quite subjective in that their claim of power-law behavior was derived solely from the observation of the behavior of distribution without any statistical testing.

Therefore, in this paper, we analyze the small-world, scale-free, and hierarchical properties of semantic networks constructed from various DSMs in a more systematic way to determine whether DSM networks exhibit the same properties as association networks. Through this network analysis, we investigate whether DSMs can provide a psychologically plausible model of semantic memory. Furthermore, we explore quantitative variations in these network properties among various DSM implementations and attempt to explain or predict them in terms of the intrinsic features and functions of DSM methods for constructing semantic spaces. In doing so, we expect network analysis to provide a new way of analyzing the properties and structures of semantic spaces created by DSMs.

In this paper, we also discuss a growing network model that produces a semantic network with the observed network properties. Steyvers and Tenenbaum [21] proposed a network model, based on the Barabási–Albert model [40] for generating scale-free networks, that simulates the scale-free and small-world properties of word association networks. Their model is characterized by the process of semantic differentiation, an extension of preferential attachment in the Barabási–Albert model. During the process of network growth a new node is connected, not to a node chosen with probability proportional to the number of connections for a node, but to the neighbors of the chosen node. Although the process of semantic differentiation enables the network model to simulate small-world properties, the authors’ evaluation of their model is not sufficient to justify the ability of the model to reproduce the scale-free property of real semantic networks. Additionally, their model does not have enough flexibility to reflect behavioral diversity of real semantic networks.

To overcome these limitations, in this paper we propose a new network model by extending the Steyvers–Tenenbaum model, and demonstrate that the proposed model provides a better explanation of the behaviors generated by various DSM implementations than the original Steyvers–Tenenbaum model. The basic idea underlying our extension of the Steyvers–Tenenbaum model is consideration of a different mechanism for network growth other than semantic differentiation (or preferential attachment) to reflect different types of semantic relations between words to be connected in a semantic network. For this purpose, we introduce the syntagmatic-paradigmatic distinction for semantic relations [41], and integrate the process of random attachment into the network model.

## Analysis of Word Association Network

Before examining the network properties of DSM networks, we confirm whether our network analysis replicates the properties of a word association network demonstrated by previous studies.

### Materials and Methods

We used the English word association norm collected at the University of South Florida (USF) [24], which has also been used in previous studies on association networks. Following these studies, we constructed a directed network as follows. First, only cue words were represented as nodes (i.e., words that appeared only as an associate were not considered). Second, two word nodes *x* and *y* were connected by a directed edge from *x* to *y*, if the word *y* was listed as an associate of cue *x* by at least two of the participants in the association experiment. We also generated an undirected network by replacing directed edges with undirected ones.

We then analyzed the properties of the word association network according to the method described below. Following existing studies on word association networks [21, 29], we restricted the analyses in this paper to the largest connected component. For the small-world property, we computed the clustering coefficient *C* and the average shortest path length *L* of a generated network, and examined whether the network satisfies *C* ≫ *C*_*random*_ and *L* ⩾ *L*_*random*_ (where *C*_*random*_ and *L*_*random*_ are the clustering coefficient and average shortest path length of the corresponding random network, respectively) [42].

For the scale-free property, we examined whether the degree distribution of the target network follows the power law *P*(*k*) ∼ *k* ^−*α*^. This was carried out not only by observing the shape of the plotted distribution, but also by applying Clauset et al.’s [43] statistical framework for testing the goodness-of-fit between the data and the power law. In their framework, the power-law distribution is fitted to the data (i.e., plotted distribution) and the power-law exponent *α* is estimated using maximum likelihood estimation. This fitting procedure assumes some lower bound *k*_*min*_ to the power-law behavior, for an empirical reason that most naturally occurring distributions only follow a power-law distribution above some lower bound. The lower bound *k*_*min*_ is identified by minimizing the Kolmogorov–Smirnov distance *D*_*KS*_ between the data and the theoretical power-law fit. In this study, to avoid generating a biased estimate by excluding many legitimate data points, we estimated the optimal *k*_*min*_ within the range *k*_*min*_ ≤ 50. The goodness-of-fit test was conducted by empirically estimating the probability *p* that *D*_*KS*_ for the observed data is smaller than that for synthetic data randomly drawn from the power-law distribution that best fits the observed data. In other words, *p* denotes the probability of obtaining the observed data under the null hypothesis that the data follow the estimated power-law model. If *p* is small, the null hypothesis is rejected. Clauset et al. [43] suggested that the power law is a plausible hypothesis if *p* > 0.1; we also used this criterion.

We also compared the estimated power-law model with two alternative models, namely the truncated power-law distribution *P*(*k*) ∼ *k* ^−*α*^e^−*λk*^, and the exponential distribution *P*(*k*) ∼ e^−*λk*^. These two distributions are frequently observed in many real systems whose degree distributions do not follow the power law [44]. Selection among these three models was conducted using 10-fold cross-validation. Each model was fitted to the training data by maximum likelihood estimation and its prediction error for the test data was estimated by a log-likelihood. Note that, for the statistical testing described here, we employed a Python package powerlaw [45].

Finally, we analyzed the hierarchical property of the network using the scaling law *C*(*k*) ∼ *k* ^−*β*^ of the local clustering coefficient *C*(*k*) of a node with degree *k* [46]. A number of studies [46–48] have demonstrated that networks involving a hierarchical topology exhibit this scaling law. However, for non-hierarchical networks, the clustering coefficient *C*(*k*) of a node is independent of its degree *k*. According to this finding, we examined the correlation between the local clustering coefficient *C*(*k*) and degree *k* on a log-log plot and a hierarchical exponent *β* for target networks. The hierarchical exponent *β* for many hierarchical networks is almost equal to 1; however, some studies [46, 49, 50] suggest that *β* = 1 is neither a sufficient nor necessary condition for a network to be hierarchical. The scaling law with *β* < 1 also indicates the hierarchical topology of the network.

### Results

Table 1 shows the network statistics for the USF association network and their corresponding random graphs. These random graphs were generated by randomly rearranging connections in the corresponding association networks. The values of *L*_*random*_ and *C*_*random*_ were computed by averaging over 10 random graphs. The association network has a small-world structure because *C* ≫ *C*_*random*_ and *L* ⩾ *L*_*random*_. This result is completely consistent with existing findings on the analysis of association networks [21, 25, 27–29].

**Table 1:** Statistics of the semantic network (*n* = 5,018) constructed from the University of South Florida (USF) association norms.

	*m*	*n*_*CC*_	⟨*k*⟩	*k*_*max*_	*D*	*L*	*L*_*random*_	*C*	*C*_*random*_
Directed	63,620	4,845	12.7	313	10	4.26	3.64	0.187	0.005
Undirected	55,236	5,018	22.0	330	5	3.04	3.03	0.187	0.005

*Note.* *n* = number of nodes; *m* = number of edges; *n*_*CC*_ = number of nodes of the largest (strongly) connected component; ⟨*k*⟩ = average node degree; *k*_*max*_ = maximum node degree; *D* = diameter of the network; *L* = average shortest path length; *L*_*random*_ = average shortest path length of the random network with the same size and density; *C* = clustering coefficient; *C*_*random*_ = clustering coefficient of the random network with the same size and density.

**Fig 1:**
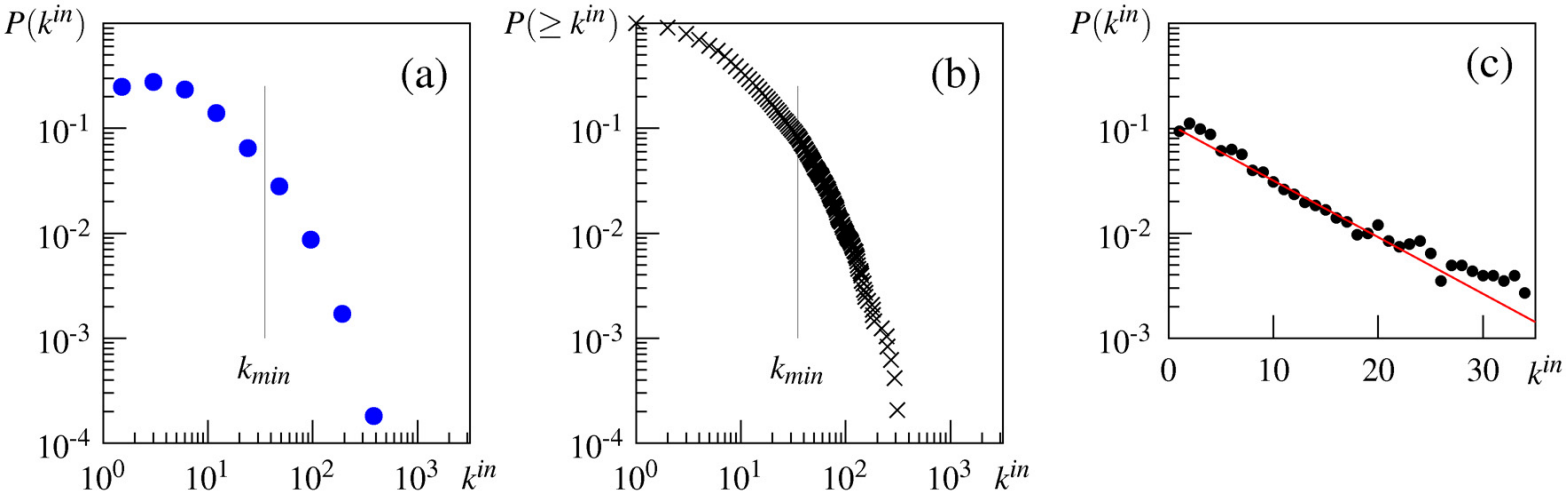
Degree distributions of the directed University of South Florida (USF) association network. (a) in-degree distribution, (b) cumulative in-degree distribution, (c) degree distribution below *k*_*min*_ on a semilogarithmic scale.

Regarding the scale-free property of the association network, Fig 1a and 1b plot the in-degree distribution of the directed association network and its cumulative distribution. These graphs show that the distributions deviate from the pure power law, as argued by Morais et al. [29]. The goodness-of-fit test for the best-fit power-law model (*α* = 2.91, *k*_*min*_ = 35) indeed ruled out the possibility of the pure power law, *D*_*KS*_ = 0.048, *p* = .01. Furthermore, the model selection procedure using 10-fold cross-validation indicated that the truncated power law (i.e., the power law with an exponential cutoff) was selected as the model that best fits the observed distribution (average log-likelihood: −176.41 for the power law, −175.60 for the truncated power law, and −176.63 for the exponential). These results are completely consistent with the findings of Morais et al. [29]. It should be noted that, in this paper, we address only the in-degree distribution to examine the scale-free property of semantic networks, because using the out-degree or the degree of the undirected network would introduce bias that stems from the task characteristics such as the number of associations [25].

Comparison of the estimated power-law exponent *α* in this and other studies also suggests that the truncated power law better describes the in-degree distribution in the USF association network. The estimated exponent is *α* = 2.91 in this study and *α* = 2.92 in that by Morais et al. [29], but these values are higher than those of other studies claiming the pure power-law fit (e.g., *α* = 1.79 [21], *α* = 2.03 [39], and *α* = 2.13 [25]). Interestingly, our estimate of *α* for the truncated power-law distribution is 1.78, which is close to their estimates of the pure power law.

We also applied the fitting procedure to the observed in-degree distribution below *k*_*min*_(= 35). This analysis is motivated by the existing finding that some semantic networks obey the power law with initial exponential decay [51]. The result is that the exponential distribution with *λ* = 0.124 best fits the data (average log-likelihood: −1375.49 for the power law, −1372.28 for the truncated power law, and −1333.30 for the exponential). Fig 1c shows that this exponential fit appears to be the case: *P*(*k*) decreases roughly linearly with the degree on the semilogarithmic (i.e., log-linear) scale, and the slope of the red line is equal to *λ* log *e*.

**Fig 2:**
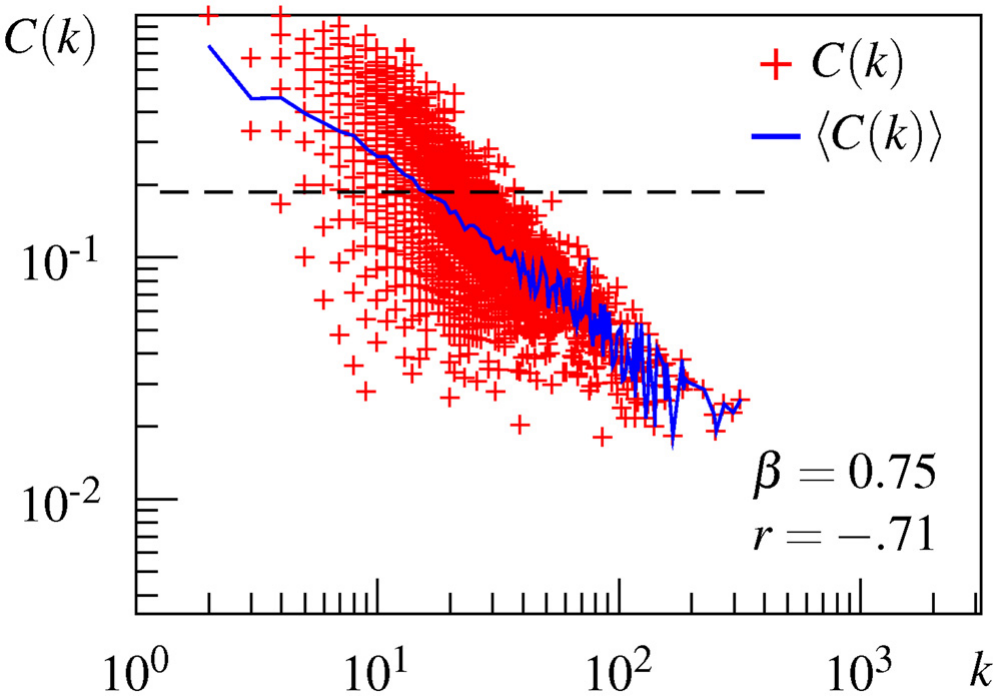
Local clustering coefficient as a function of the node degree for the USF network. Red plots denote the local clustering coefficient of an individual node, the blue line connects the average local clustering coefficient with the same degree, and the dashed line denotes the clustering coefficient *C*. *β* = the hierarchical exponent, *r* = the correlation coefficient between *C*(*k*) and *k*.

Finally, to examine whether a hierarchical structure is involved in the association network, a local clustering coefficient *C*(*k*) is plotted against the degree *k* on a logarithmic scale, as shown in Fig 2. This plot is derived from the undirected version of the largest strongly connected component of the directed USF network. Fig 2 shows that the USF association network has a hierarchical structure. The local clustering coefficient *C*(*k*) of a node is negatively correlated with its degree *k* on a log-log plot (*r* = −.71). The dependency is linearly fitted in a log-log plot, as indicated by the blue line connecting the average of local clustering coefficients across points with the same degree. The estimated slope *β* = 0.75 is smaller than 1, but, interestingly, the maximum value of *C*(*k*) follows the line with *β* = 1.02. This result indicates that hierarchical modularity exists in the USF association network.

## Analysis of DSM Networks

### Materials and Methods

To compare DSM networks directly with the USF association network, we used only the cue words of the USF association norm when creating the DSM networks. As a corpus for DSMs, we used the written and non-fiction parts of the British National Corpus, comprising 491,106 documents, 73,422 distinct words, and 4,702 cue words.

We created a semantic network from a given semantic space by first computing the cosine similarity between pairs of words and then determining local neighborhoods using cosine similarity. Local neighborhoods were determined by two methods, namely the *k*-nn method and the *cs*-method. The *k*-nn method was used by Steyvers and Tenenbaum in their study [21], while the *cs*-method was devised in this study. Although both methods create directed edges from each word to its nearest neighbors, they differ in the way that the number of nearest neighbors is determined for each word. In the *k*-nn method, the number of neighbors for word *w*_*i*_ is set to the number of associates of that word in the USF association norms. Therefore, the out-degree distribution of a DSM network is identical to the out-degree distribution of the USF association network. In the *cs*-method, the number of neighbors for word *w*_*i*_ is determined to be the smallest $k=|V_{i}^{N}|$ such that the cumulative similarity ratio of $V_{i}^{N}$ exceeds the threshold *R*:

$$\begin{array}{c} \frac{\sum_{w_{j}\in V_{i}^{N}}cos\left(w_{i},w_{j}\right)}{\sum_{w_{j}\in V}cos\left(w_{i},w_{j}\right)}\;>\;R \end{array}$$ (1)

where $V_{i}^{N}$ is the set of *k* nearest neighbors of *w*_*i*_, *V* is the set of all words except *w*_*i*_, and *cos*(*w*_*i*_, *w*_*j*_) is the cosine similarity between words *w*_*i*_ and *w*_*j*_. The threshold *R* was determined such that the created DSM network has the same ⟨*k*⟩ as in the directed association network. Note that Steyvers and Tenenbaum [21] also used the *ɛ*-method, in which local neighborhoods are computed by thresholding the cosine similarity; that is, any pair of words whose cosine value is equal to or higher than threshold *ɛ* is connected by an undirected edge. However, the symmetric nature of this method is not appropriate for modeling semantic knowledge underlying word association. In human word association, having word *x* as an associate of cue word *y* does not imply that *y* is an associate of *x*, but the *ɛ*-method cannot capture this difference. Therefore, we did not use the *ɛ*-method in this paper.

In the DSM framework, semantic spaces are constructed according to the following three steps [32].

1. **Initial matrix construction:** A word-context frequency matrix **A** is constructed with *n*_*w*_ rows for words and *n*_*c*_ columns for the contexts. An element *a*_*ij*_ of **A** is the frequency *f*_*ij*_ of word *w*_*i*_ in a context *c*_*j*_; hence, the *i*-th row corresponds to the initial word vector for the *i*-th word *w*_*i*_.
2. **Weighting:** The elements of the matrix **A** are weighted.
3. **Smoothing:** The dimension *n*_*c*_ of the row vectors of **A** is reduced to *n*_*r*_.

The notion of context *c*_*j*_ in Step 1 can generally be classified into two types: “documents as contexts” and “words as contexts.” For a documents-as-contexts (or word-document) matrix, the frequency *f*_*ij*_ for **A** is the number of times that word *w*_*i*_ occurs in document *d*_*j*_. For a words-as-contexts (or word-word) matrix, the frequency *f*_*ij*_ is the number of times that word *w*_*i*_ cooccurs with word *w*_*j*_ within a certain range such as a window of some words. In this paper, we used both types of contexts to construct the initial matrix, and a context window of size two (i.e., two words on either side of the target word) was used to generate a word-word matrix.

In Step 2, we employed two popular weighting methods, tf-idf and ppmi. In the tf-idf weighting scheme, the weight is calculated as the product of the local weight based on the term frequency and the global weight based on the inverse document frequency or entropy. In this paper, we used the following function (i.e., the product of the logarithm of the word frequency and the entropy) [52, 53]:

$$\begin{array}{ccc} a_{ij} & = & \log\left(f_{ij}+1\right)*(1+\frac{\sum_{k=1}^{n_{c}}P_{ik}\log P_{ik}}{\log n_{c}}) \end{array}$$ (2)

$$\begin{array}{ccc} P_{ij} & = & \frac{f_{ij}}{\sum_{k=1}^{n_{c}}f_{ik}} \end{array}$$ (3)

where *a*_*ij*_ is a weighted element for word *w*_*i*_ in context *c*_*j*_. In the ppmi weighting scheme, weight *a*_*ij*_ is calculated by pointwise mutual information defined in Eq 5, and negative values are replaced with zero [34].

$$\begin{array}{ccc} a_{ij} & = & \left\{ \begin{array}{cc} \;\;pmi_{ij} & \;\text{(if}\;pmi_{ij}>0\text{)}\\ \;\;0 & \;\left(otherwise\right) \end{array}\right. \end{array}$$ (4)

$$\begin{array}{ccc} pmi_{ij} & = & \log\frac{p_{ij}}{p_{i*}p_{*j}}\\ p_{ij} & = & \frac{f_{ij}}{\sum_{i=1}^{n_{w}}\sum_{j=1}^{n_{c}}f_{ij}}\\ p_{i*} & = & \frac{\sum_{j=1}^{n_{c}}f_{ij}}{\sum_{i=1}^{n_{w}}\sum_{j=1}^{n_{c}}f_{ij}}\\ p_{*j} & = & \frac{\sum_{i=1}^{n_{w}}f_{ij}}{\sum_{i=1}^{n_{w}}\sum_{j=1}^{n_{c}}f_{ij}} \end{array}$$ (5)

In Step 3, matrix smoothing was conducted using singular value decomposition (SVD). In this study, we set *n*_*r*_ = 300, which is also used in typical applications of LSA.

Using the methods explained above, we created 24 DSM networks from all possible combinations of the two methods for determining neighborhoods (*k*-nn or *cs*-method), two initial matrices (word-document or word-word), three weighting options (tf-idf, ppmi, or unweighted), and two smoothing options (SVD or unsmoothed). Note that LSA, which was used by Steyvers and Tenenbaum [21] to construct semantic spaces, corresponds to the combination of a word-document matrix, tf-idf weighting, and SVD smoothing. We then analyzed the properties of these DSM networks according to the method described in the Analysis of Word Association Network section.

### Results for Small-world Property

**Fig 3:**
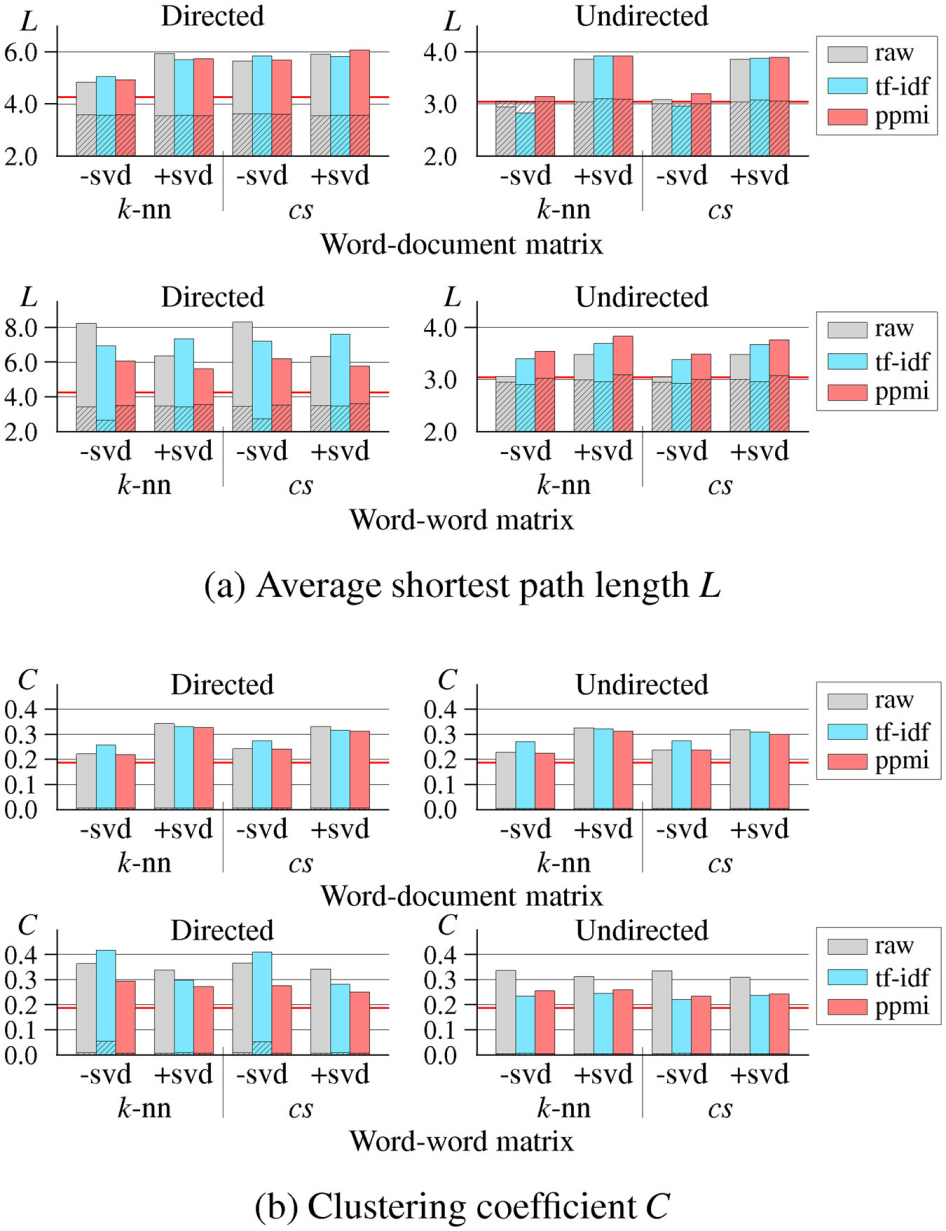
Average shortest path length *L* and clustering coefficient *C* of all distributional semantic model (DSM) networks. The hatched part of each bar graph represents *L*_*random*_ and *C*_*random*_.

Table 2 shows the network statistics for some representative examples of DSM networks. (Statistics for all 24 DSM networks are given in S1 Table). Fig 3 depicts the clustering coefficient and shortest path length of all 24 DSM networks and their corresponding random graphs. These random graphs were generated by randomly rearranging connections in the corresponding semantic networks. The values of *L*_*random*_ and *C*_*random*_ were computed by averaging over ten random graphs. Although there are slight differences in these variables among the DSM networks, these results clearly indicate that all the DSM networks have a small-world structure (i.e., high clustering coefficient, small shortest path length, and high connectivity).

**Table 2:** Statistics for some representative examples of distributional semantic model (DSM) networks (*n* = 4,702).

	*m*	*n*_*CC*_	⟨*k*⟩	*k*_*max*_	*D*	*L*	*L*_*random*_	*C*	*C*_*random*_
Word-document matrix, unweighted, unsmoothed, *k*-nn method									
Directed	60,262	4,519	12.8	1,894	13	4.84	3.59	0.222	0.006
Undirected	49,274	4,702	21.0	2,044	5	2.94	3.06	0.228	0.005
Word-document matrix, tf-idf, unsmoothed, *k*-nn method									
Directed	60,252	4,403	12.8	2,193	14	5.05	3.58	0.257	0.006
Undirected	50,219	4,702	21.4	2,456	5	2.83	3.04	0.270	0.005
Word-document matrix, unweighted, smoothed, *cs*-method									
Directed	59,613	3,897	12.6	176	23	5.92	3.55	0.331	0.006
Undirected	49,925	4,702	21.2	201	8	3.86	3.04	0.318	0.005
Word-document matrix, tf-idf, smoothed, *cs*-method									
Directed	59,613	4,156	12.6	296	27	5.83	3.58	0.317	0.006
Undirected	48,622	4,702	20.7	321	8	3.88	3.07	0.308	0.005
Word-word matrix, unweighted, unsmoothed, *k*-nn method									
Directed	60,251	3,296	13.2	831	24	8.25	3.43	0.363	0.008
Undirected	55,785	4,702	23.7	1122	6	3.06	2.95	0.336	0.005
Word-word matrix, ppmi, unsmoothed, *k*-nn method									
Directed	60,250	3,748	13.1	199	26	6.08	3.50	0.294	0.007
Undirected	51,316	4,702	21.8	235	7	3.54	3.02	0.255	0.005
Word-word matrix, unweighted, smoothed, *cs*-method									
Directed	59,613	3,563	12.8	388	18	6.35	3.50	0.342	0.007
Undirected	52,700	4,702	22.4	473	7	3.48	3.00	0.308	0.005
Word-word matrix, ppmi, smoothed, *cs*-method									
Directed	59,613	4,474	12.7	106	19	5.77	3.60	0.251	0.006
Undirected	48,504	4,702	20.6	108	8	3.76	3.07	0.242	0.005

*Note.* *n* = number of nodes; *m* = number of edges; *n*_*CC*_ = number of nodes of the largest (strongly) connected component; ⟨*k*⟩ = average node degree; *k*_*max*_ = maximum node degree; *D* = diameter of the network; *L* = average shortest path length; *L*_*random*_ = average shortest path length of the random network with the same size and density; *C* = clustering coefficient; *C*_*random*_ = clustering coefficient of the random network with the same size and density.

### Results for Scale-free Property

In this section, we first discuss the overall results of the statistical analysis (i.e., the goodness-of-fit test for the power law and model selection by 10-fold cross-validation) for all the DSM networks. Thereafter, we provide a more detailed analysis of the scale-free property of DSM networks by observing their in-degree distributions. We first compare the in-degree distributions for two types of initial matrices and then examine the effect of weighting and smoothing on power-law behavior.

#### Overall result

Table 3 shows the results of statistical testing of all the DSM networks. The symbols for each code are divided into two parts. The first symbol (+ or -) denotes the result of the goodness-of-fit test for the power law, where + denotes that the pure power law is a plausible hypothesis for the data and - indicates that it can be ruled out. The subsequent symbols (any of P, T, and E) denote the most appropriate distributions selected by 10-fold cross-validation. Symbols P, T, and E denote, respectively, that the power law, truncated power law, and exponential are selected as the distribution with the best fit. If multiple models have equal average log-likelihoods rounded to one decimal place, all the corresponding symbols are listed in the order given above. Details of the statistical tests are given in S2 Table.

**Table 3:** Summary of statistical testing for power-law behavior of in-degree distributions of directed DSM networks.

matrix / method	Unsmoothed			Smoothed		
	raw	tf-idf	ppmi	raw	tf-idf	ppmi
word-document / *k*-nn	+T	+T	+PT	+PT	+P	+PTE
word-document / *cs*	+PT	+PT	+PT	+PT	+P	+T
word-word / *k*-nn	+T	-TE	-T	+T	+PT	+T
word-word / *cs*	+T	-T	+PT	+T	+PT	+PT

*Note.* Codes used in this table start with a symbol denoting the result of the goodness-of-fit test for the power law, followed by one to three symbols denoting the result of model selection by 10-fold cross-validation. Symbol + denotes that the power law fits the data, while symbol - denotes that the power law is ruled out. Symbols P, T, and E denote that the power law, truncated power law, and exponential, respectively, are selected as the best fitting distribution. When more than one symbol is presented as the result of model selection, it means that the average log-likelihoods of these models, rounded to one decimal place, are equal.

Code +P indicates that a pure power-law distribution is definitely the most appropriate, while codes +PT and +PTE indicate that a pure power law is very likely to be most appropriate. Conversely, codes -T, -TE, and +T indicate that a truncated power-law degree distribution is most appropriate, although in the case of -TE, an exponential distribution cannot be ruled out. Other possible code patterns are not obtained for DSM networks. Note that the test result for the USF association network is coded as -T, which favors a truncated power law.

Overall, Table 3 shows that all the DSM networks exhibit a power-law or a truncated power-law distribution; 13 networks are coded as +P, +PT, or +PTE in favor of the pure power law, and the remaining 11 networks are coded as -T, -TE, or +T in favor of the truncated power law. In addition, we also analyzed the observed in-degree distribution below the lower bound *k*_*min*_ using the same procedure as in the analysis of the word association network. The result is that, for all 24 DSM networks, the exponential distribution is a significantly better fit for the data below *k*_*min*_ than both the pure and truncated power laws. These results provide direct evidence against Steyvers and Tenenbaum’s [21] argument that LSA networks do not have a power-law distribution, and thus, LSA cannot provide a plausible model of semantic memory. Instead, the results indicate that a DSM has the ability to produce semantic networks with a degree distribution that is the same as or similar to that of the association network. Hence, network analysis also confirms that a DSM can provide a psychologically plausible framework for modeling human semantic memory.

#### Comparison of initial frequency matrix

**Fig 4:**
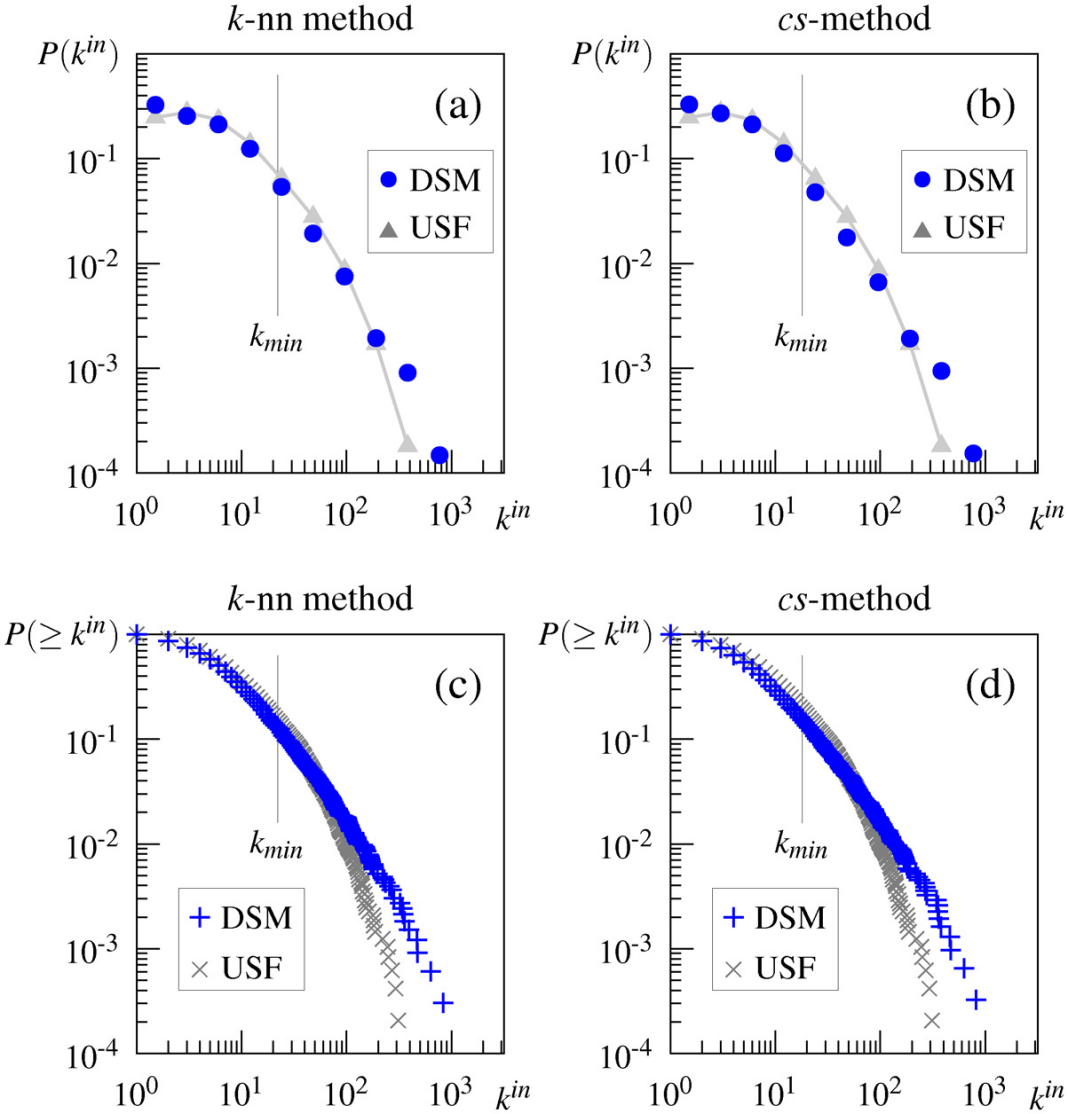
In-degree and cumulative in-degree distributions of the DSM network generated from the initial word-word matrix. (a) in-degree distribution of the DSM networks generated by the *k*-nn method, (b) in-degree distribution of the DSM networks generated by the *cs*-method, (c) cumulative in-degree distribution of the DSM networks generated by the *k*-nn method, (d) cumulative in-degree distribution of the DSM networks generated by the *cs*-method. DSM = DSM network, USF = USF association network.

As shown in Fig 4 (and in the second column +T of Table 3), the in-degree distributions of the DSM networks generated from the initial word-word matrix follow the truncated power-law degree distribution. These distributions are similar to, but less truncated than the distribution of the USF association network.

**Fig 5:**
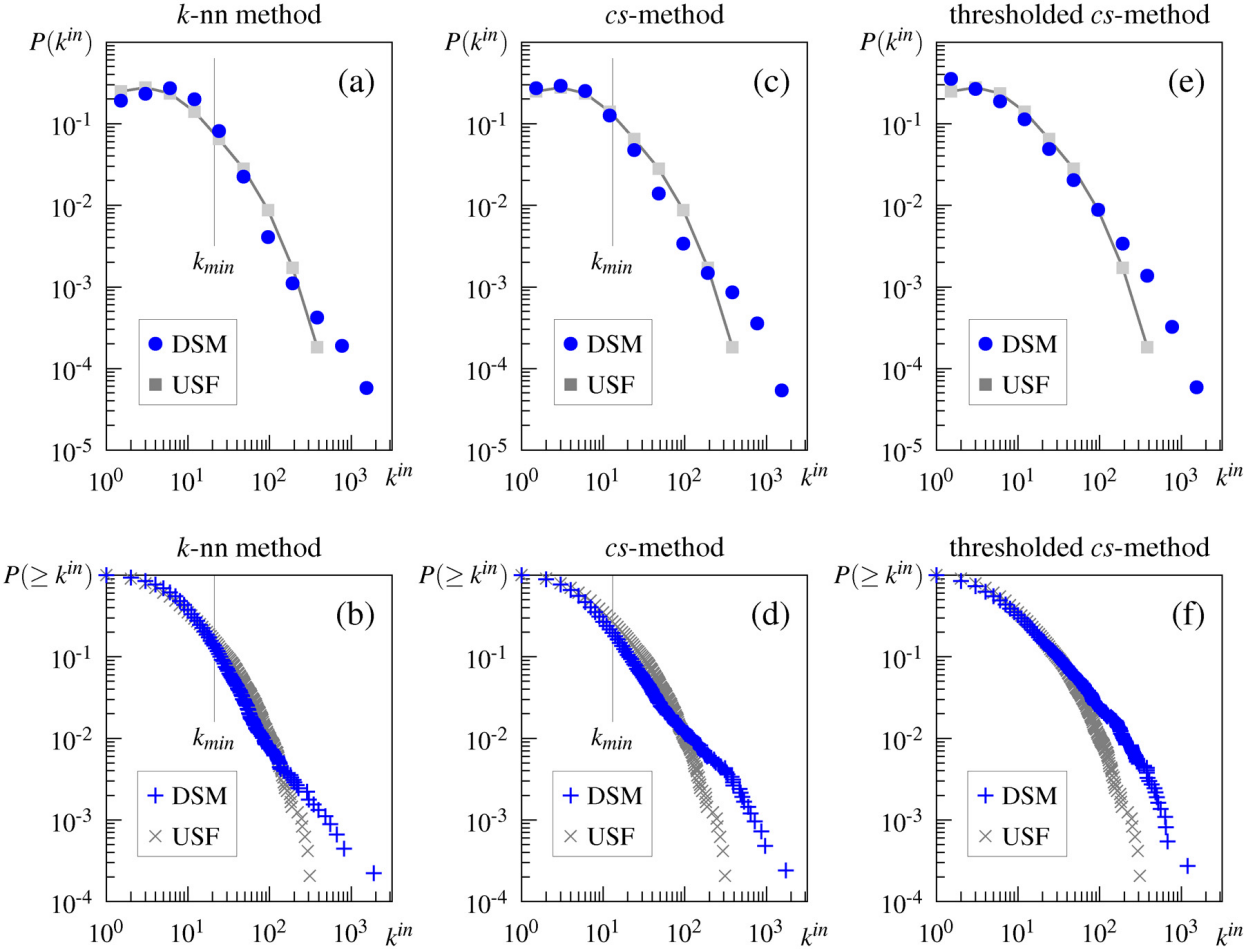
In-degree and cumulative in-degree distributions of the DSM network generated from the initial word-document matrix. (a) in-degree distribution of the DSM networks generated by the *k*-nn method, (b) cumulative in-degree distribution of the DSM networks generated by the *k*-nn method, (c) in-degree distribution of the DSM networks generated by the *cs*-method, (d) cumulative in-degree distribution of the DSM networks generated by the *cs*-method, (e) in-degree distribution of the DSM networks generated by the thresholded *cs*-method, (f) cumulative in-degree distribution of the DSM networks generated by the thresholded *cs*-method. DSM = DSM network, USF = USF association network.

In contrast, as shown in Fig 5a–5d, the shape of the degree distributions of the DSM networks generated from the word-document matrix differs from both the distribution of the association network and the pure power law; the distributions decay exponentially for small *k*, but the decay suddenly decreases in a linear fashion, which is unlikely to be observed in real-world systems. Although the second column of Table 3 indicates that a pure or truncated power law is favored over an exponential one, the shape obviously differs from these three distributions.

One probable explanation for these unnatural distributions is that, owing to its high sparsity, the word-document matrix may generate inappropriate DSM networks, whereby word pairs with very low cosine similarity are connected by edges. In general, a word-document matrix is sparser than a word-word matrix generated from the same corpus; the percentage of zero elements was 99.36% for the word-document matrix used in this study, and 78.84% for the word-word matrix. The high sparseness of the word-document matrix leads many word pairs to have very low cosine similarity. Indeed, in the word-document-based DSM network by the *cs*-method, about half the word pairs connected by an edge had a cosine of 0.05 or less. These low-cosine word pairs must be chosen for an edge such that the DSM network has the same average degree as the association network. It is appropriate for word pairs that are somewhat related to be connected by an edge. However, despite being semantically unrelated, many other pairs are chosen because they have low cosine values by chance. In this case, more frequent words tend to have a low, but non-zero cosine similarity to more unrelated words, because they have more non-zero elements in their vector representation. The fatter tail observed in the degree distribution for the word-document matrix may be a consequence of this frequency effect; some frequent words are connected by a large number of edges. Hence, if a network is created by thresholding the cosine similarity of word pairs, it is expected that its degree distribution approaches the (truncated) power law. Indeed, as shown in Fig 5e and 5f, the degree distribution of the network we created using the *cs*-method by limiting word pairs to be joined by an edge to those with the cosine greater than 0.05, followed the truncated power law. The same result was obtained in networks created using the *k*-nn method.

#### Effect of weighting

**Fig 6:**
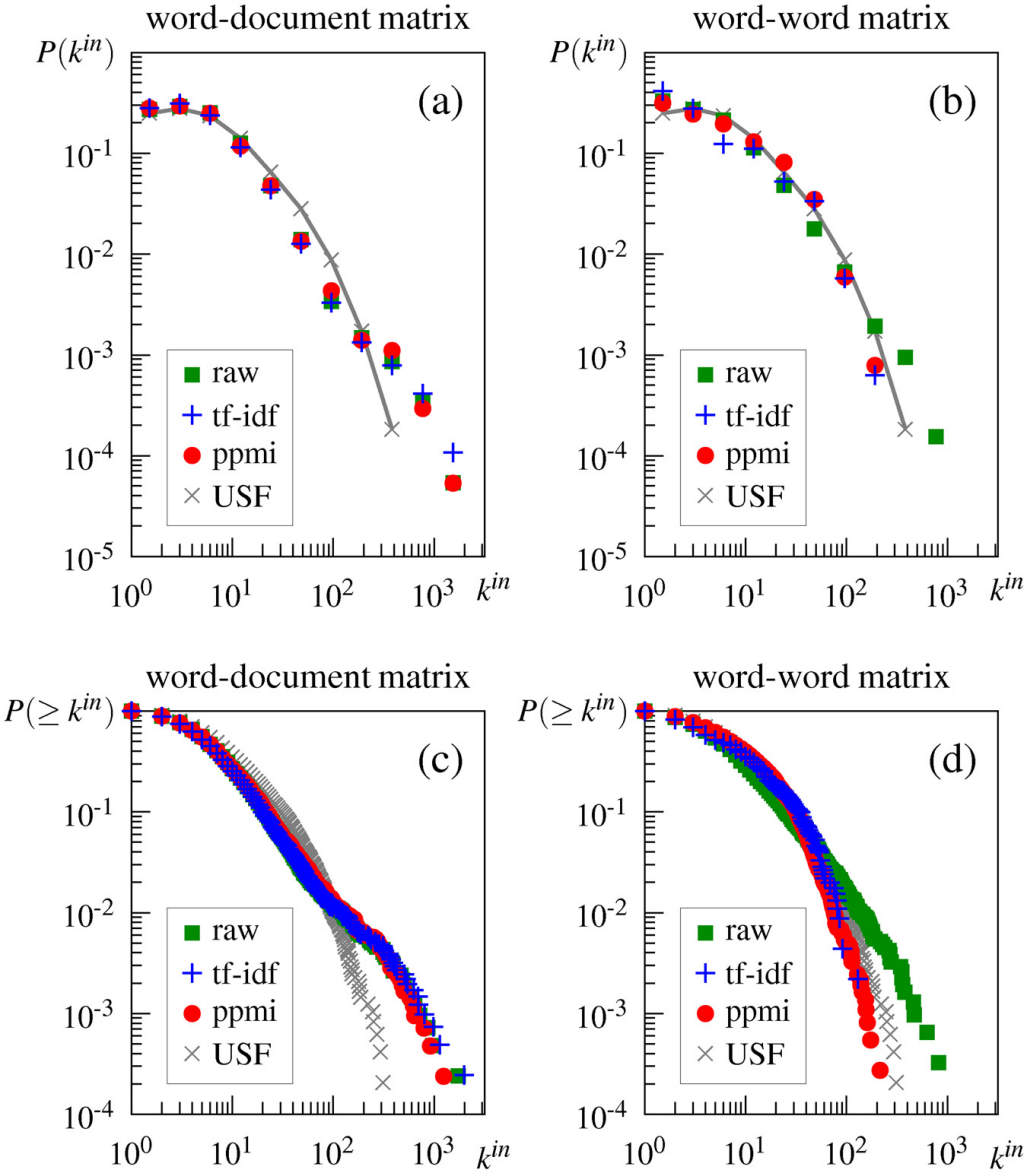
In-degree and cumulative in-degree distributions of the DSM networks generated from different weighting schemes by the *cs*-method. (a) in-degree distribution of DSM networks generated from the word-document matrix, (b) in-degree distribution of DSM networks generated from the word-word matrix, (c) cumulative in-degree distribution of DSM networks generated from the word-document matrix, and (d) cumulative in-degree distribution of DSM networks generated from the word-word matrix. raw = unweighted DSM, tf-idf = DSM with tf-idf weighting, ppmi = DSM with ppmi weighting, USF = USF association.

Weighting does not change the unnatural degree distribution observed in the word-document-based networks, as shown in Fig 6a–6c, and in the third and fourth columns of Table 3. (Note that Fig 6 shows only the distributions for DSM networks generated by the *cs*-method. The results of the DSM networks generated by the *k*-nn method are shown in S1 Fig, but do not differ from the results of the *cs*-method.)

However, in the case of the word-word matrix, weighting alters the degree distribution; both tf-idf and ppmi weighting change the degree distribution into a more truncated form (i.e., with a sharper cutoff) whose curve is more similar to that of the association network, as shown in Fig 6b–6d. Table 3 (i.e., codes -T and -TE in the third and fourth columns) shows that the distributions are no longer plausible power laws and follow the truncated power law, although a pure power law is most plausible for the network generated by the *cs*-method with ppmi weighting.

The obtained result can be explained by the role of weighting in constructing word vectors. The role of weighting is to assign a greater weight to unexpected events, that is, contexts that are more significantly associated with to a target word, and a smaller weight to expected events [32]. For example, frequent words are likely to occur in a context even if they are not semantically associated with that context. Weighting downplays these expected events to compute the similarity between two word vectors correctly. Conversely, weighting highlights those contexts in which a word occurs more often than would be expected by chance. This function of weighting also makes word vectors semantically more appropriate, and as a result, semantically related words are more likely to be connected by an edge in the DSM networks generated from the word-word matrix. However, weighting does not work as a method for reducing the sparsity of a matrix; instead, sparsity is often increased. Because the sparsity of the word-document matrix is the main cause of the unnatural degree distribution of its DSM networks as mentioned previously, weighting does not remedy the problem and the DSM networks generated from the weighted word-document matrix still exhibit the unnatural distribution.

#### Effect of smoothing

**Fig 7:**
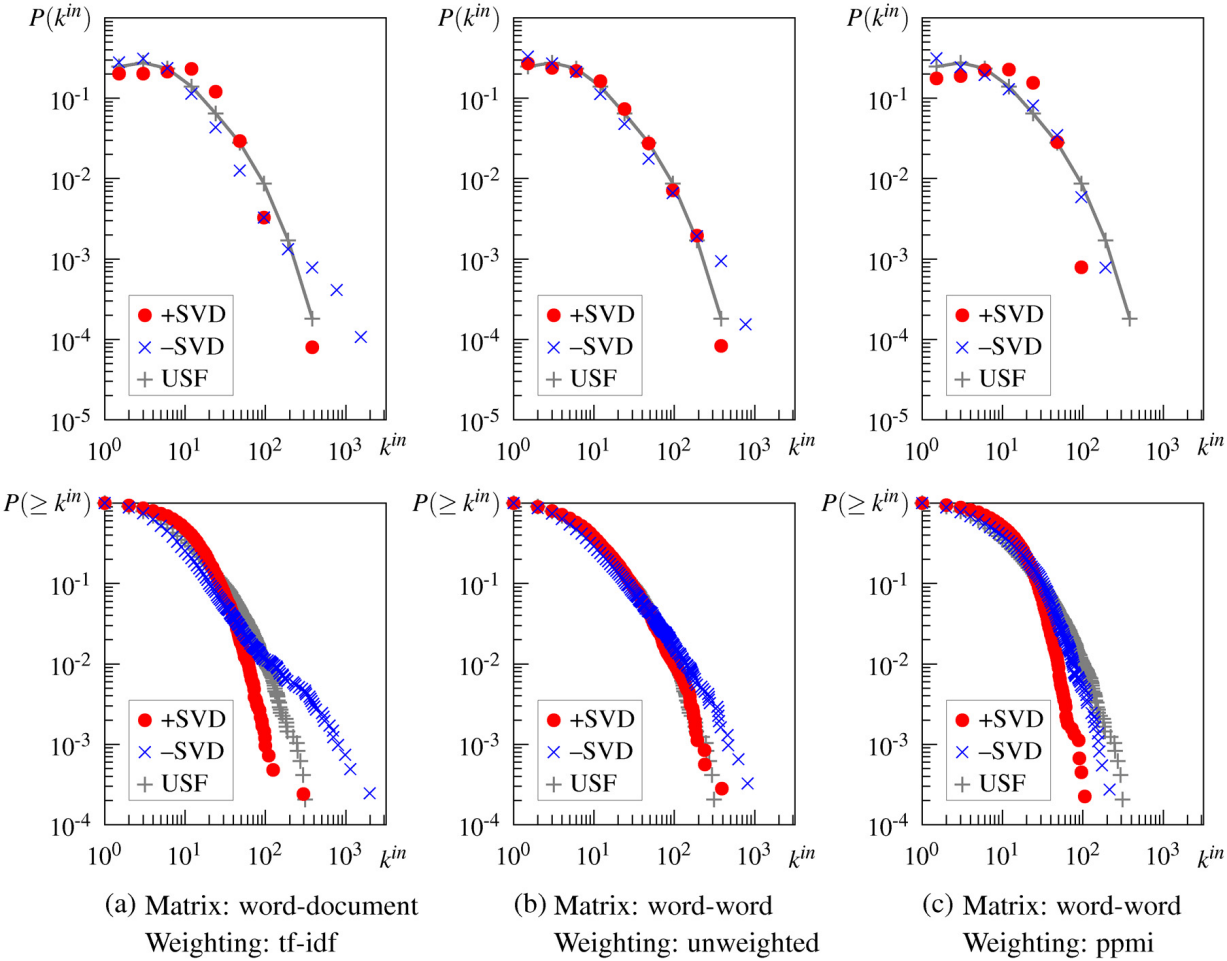
In-degree and cumulative in-degree distributions of some DSM networks generated after and before singular value decomposition (SVD) smoothing by the *cs*-method. +SVD = smoothed DSM, −SVD = unsmoothed DSM, USF = USF association.

SVD smoothing alters the degree distributions of DSM networks with both types of context. The smoothed word-document matrix yields a scale-free network whose degree distribution follows the pure power law above some lower bound, although it exhibits slightly unusual behavior for small *k*, as shown in Fig 7a. (Fig 7 shows the result for the network generated from the tf-idf-weighted word-document matrix by the *cs*-method, but this does not differ from the results of the unweighted and ppmi-weighted matrix and those of the networks generated by the *k*-nn method. For these results, see S1 Fig)

In the case of the word-word matrix, SVD smoothing also affects the DSM network such that its degree distribution takes a more truncated form. As a result, the DSM network based on the unweighted word-word matrix follows almost the same truncated power-law distribution as the USF association network, as shown in Fig 7b. The DSM networks based on the weighted matrix exhibit a power-law distribution above a high lower bound, as shown in Fig 7c and indicated by +PT in the last two columns of Table 3.

The obtained result is likely due to the functions of SVD smoothing, namely sparsity reduction and discovery of latent meanings. For the word-document matrix, its sparsity is greatly reduced by SVD smoothing and the resulting DSM network exhibits a power-law degree distribution in the same way as other DSM networks. For the word-word matrix, the structure of DSM networks may be influenced by latent meanings that cannot be directly derived from the unsmoothed matrix but can be discovered by SVD smoothing. Despite being actually semantically related, two words that do not cooccur in any context are computed as completely unrelated by the unsmoothed DSM. Using SVD smoothing to overcome this difficulty, semantically related words are likely to be computed correctly and the resulting DSM network captures human semantic knowledge more accurately.

#### Summary

According to the behavior of the USF association network, the power-law behavior of degree distributions of DSM networks can be categorized into the following four types.

Table 4 shows the result of categorization of all DSM networks.

- Moderate truncated power law (Type M): This type of distribution follows the truncated power law exhibited by the USF association network. In other words, Type M distributions are highly similar or almost identical to the distribution of the USF association network. The distribution of the DSM network for the unweighted and smoothed word-word matrix (the red plot in Fig 7b) is a typical example of Type M.
- Less truncated power law (Type L): This type of distribution follows a less truncated power law (or seemingly moderate power law) than the distribution of the USF network. The distribution of the DSM network for the unweighted and unsmoothed word-word matrix (Fig 4) is a typical example of Type L.
- Steep power law (Type S): This type of distribution follows a power law above a lower bound, but its power-law slope is steeper than those of other classes of distributions (i.e., the exponent *α* > 4). Distributions of the DSM networks based on the tf-idf-weighted and smoothed word-document matrix (the red plot in Fig 7a) and the ppmi-weighted and smoothed word-word matrix (the red plot in Fig 7c) are typical examples of Type S.
- Power law with an unnatural, fat tail (Type F): This type of distributions seems to follow a power law, but exhibits an unnatural, fat tail for large *k*, owing to the data sparseness of the initial DSM matrix. The distribution of the DSM network for the unweighted and unsmoothed word-document matrix (Fig 5a–5d) is a typical example of Type F.

**Table 4:** Type of power-law behavior of in-degree distribution of DSM networks.

matrix / method	Unsmoothed			Smoothed		
	raw	tf-idf	ppmi	raw	tf-idf	ppmi
word-document / *k*-nn	F	F	F	S	S	S
word-document / *cs*	F	F	F	S	S	S
word-word / *k*-nn	L	M	M	M	S	S
word-word / *cs*	L	M	M	M	S	S

*Note.* L: less truncated power law, M: moderate truncated power law similar to that of the USF network, S: power law with a steep power-law slope, F: power law with an unnatural, fat tail.

As shown in Table 4, the behavior of the degree distribution of DSM networks differs greatly between the word-document matrix and the word-word matrix, although it does not depend on the methods for weighting (tf-idf, ppmi) and neighborhood determination (*k*-nn, *cs*). Owing to its data sparseness, the unsmoothed word-document matrix creates semantic networks of Type F. SVD smoothing compensates for the lack of information due to data sparseness, thus leading to scale-free DSM networks of Type S. On the other hand, the unsmoothed word-word matrix creates a network of Type M or Type L, which exhibits a truncated power-law degree distribution. When DSMs are smoothed, their networks exhibit more truncated distributions, becoming Type S or Type M networks.

### Results for Hierarchical Property

**Fig 8:**
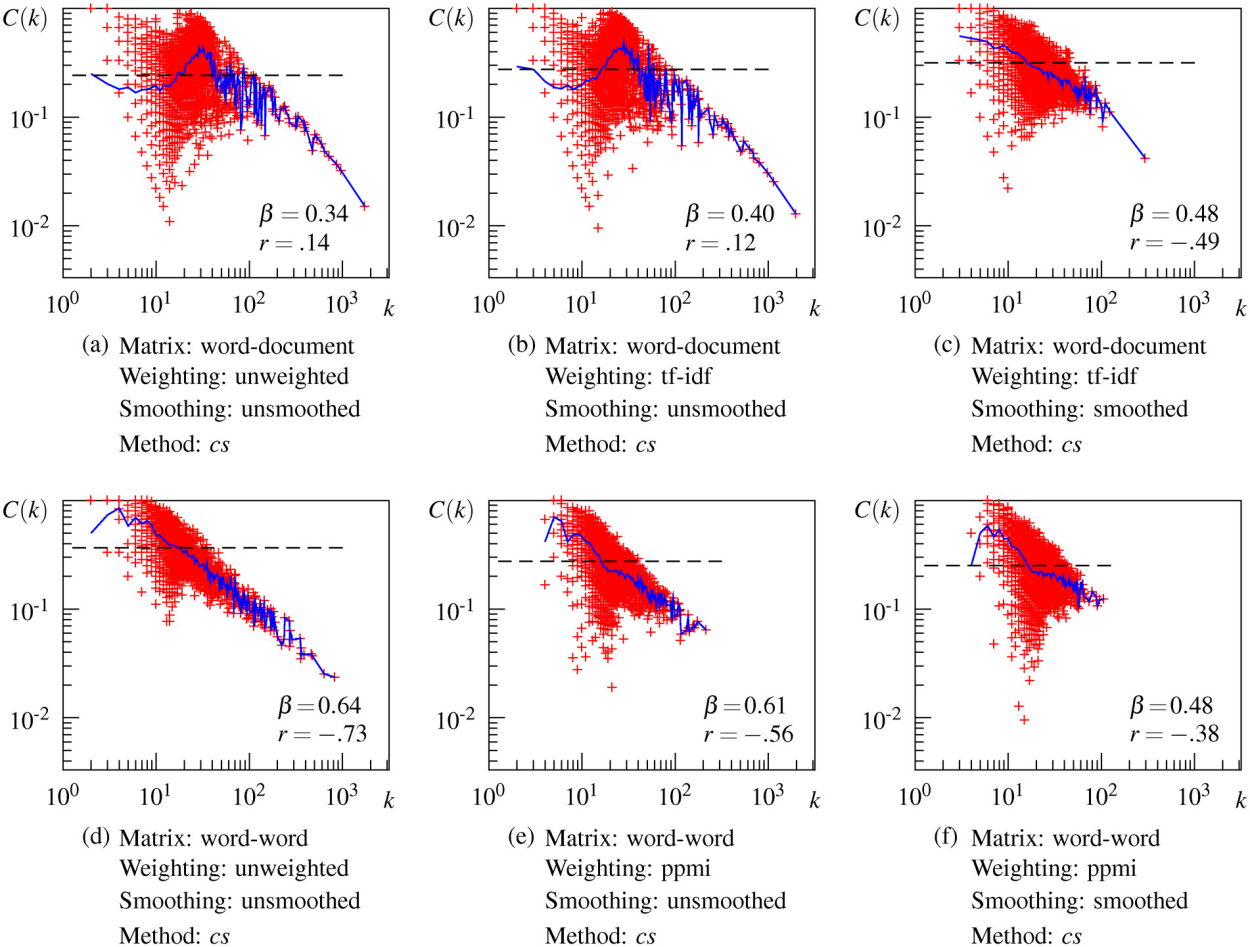
Local clustering coefficient as a function of the node degree for some representative DSM networks. Red plots denote the local clustering coefficient of an individual node, the blue line connects the average local clustering coefficient with the same degree, and the dashed line denotes the clustering coefficient *C*.

Fig 8 shows the correlation between the local clustering coefficient *C*(*k*) and degree *k* on a log-log plot for some DSM networks. (The correlation for all 24 DSM networks is depicted in S2 Fig)

Overall, DSM networks show different patterns of dependency between *C*(*k*) and *k* depending on the type of context for the initial matrix. The DSM network generated from the initial word-document matrix does not show a meaningful correlation between *C*(*k*) and *k* (Fig 8a), although the plot seems to follow a power law above degree *k* ∼ 10^2^. The result remains unchanged when the word-document matrix is weighted by tf-idf, as shown in Fig 8b. These results indicate that unsmoothed word-document-based DSM networks are not organized hierarchically, or at least do not have the same level of hierarchy as the USF association network. In contrast, the DSM network generated from the initial word-word matrix exhibits hierarchical modularity in the same way as the USF association network (see Fig 8d). The correlation between *C*(*k*) and *k* is negative and strong (*r* = −.73) and the average *C*(*k*) follows the power law, although the hierarchical exponent *β* is less than that of the USF association network. The ppmi weighted word-word matrix also generates a hierarchically structured network as shown in Fig 8e. These results indicate that unsmoothed word-word-based DSM networks are organized hierarchically.

SVD smoothing works differently for word-document-based and word-word-based networks. When the word-document matrix is smoothed, *C*(*k*) is moderately correlated with *k* (*r* = −.49) and the average *C*(*k*) follows a power law with a small exponent *β*, thus suggesting that a hierarchical structure seems to emerge in the resulting DSM network (Fig 8c). In contrast, when the word-word matrix is smoothed, the correlation between *C*(*k*) and *k* becomes weaker and *C*(*k*) no longer follows a power law in the resulting DSM network (Fig 8f). This result suggests that the hierarchical modularity tends to collapse partially by smoothing the word-word matrix. As a result of smoothing, DSM networks generated by both types of matrices exhibit a similar hierarchical structure.

Despite these differences in power-law behavior of *C*(*t*) among the DSM networks, the *C*(*t*) plots for all DSM networks share some characteristics. The local clustering coefficient *C*(*k*) diverges around the *k* ∼ 10^1^ region and the average of this region characterizes the clustering coefficient *C* of the whole network, as indicated by the intersection of the dashed line with the blue line in this region in Fig 8. The wider divergence of *C*(*k*) in this region implies that some nodes are part of densely interlinked clusters, while other nodes are outside of these clusters despite having a considerable number of connections. This is the main cause of non-hierarchy in the word-document-based DSM networks shown in Fig 8a and 8b. At the same time, all the networks share the behavior of the *C*(*k*) curve, that is, for nodes with large *k* above this region, *C*(*k*) decreases linearly with *k*. Neighbors of hub nodes (i.e., highly connected nodes) have a small chance of being linked to one another, and thus hub nodes connect distant clusters and other groups of nodes that do not form clusters. This implies that hub nodes occur at a higher level in the hierarchical structure of the network. From these common characteristics, we can reasonably assume that DSM networks consist of a mixture of hierarchical modules and a non-hierarchical set of nodes. The observed difference in power-law behavior of *C*(*k*) among the DSM networks may reflect the relative proportion of nodes in the hierarchical modularity. What these results imply about the semantic properties involved in the semantic network is explained in the next section.

## Dynamics of DSM Networks

In the last section, we demonstrated that, in general, some DSMs can generate semantic networks with the same scale-free and hierarchical properties as the association network, and in particular, DSM networks have different degree distributions and hierarchical structures depending on the way their semantic spaces have been constructed. Two questions that naturally arise and must be addressed are how the structure of these semantic networks emerges and what structural factors govern the different behaviors of DSM networks. In this section, we attempt to provide a probable answer to each of these questions in terms of semantic relations between connected word nodes. We propose that distinction of the two types of semantic relations, namely, syntagmatic and paradigmatic relations, is a key factor in explaining the structure and dynamics of semantic networks. The distinction of semantic relations has not been addressed in existing studies on network analysis of semantic networks; thus, we argue that this study can shed new light on the structure and dynamics of semantic networks.

### Semantic Relation

The basic premise of semantic networks is that two word nodes are connected by an edge if these words are semantically related. In a word association network [21, 29], semantic relatedness between two words is determined according to whether one word is an associate of the other word. In a word cooccurrence network [15, 54], it is determined according to the cooccurrence frequency in the text. In a DSM network, it is computed as the cosine similarity between two word vectors in a semantic space. Despite these different approaches to the computation of semantic relatedness, the semantic relation that exists between words determined to be semantically related is the same and can be classified into a few types. However, existing network analysis studies on semantic networks do not focus on the type of semantic relations.

In lexical semantics [41], semantic relations can be classified into two types: *syntagmatic* and *paradigmatic* relations. The syntagmatic-paradigmatic distinction originates back to Ferdinand de Saussure, and has been addressed by a number of studies on word associations [25, 55]. Two words are syntagmatically related if they cooccur more often than would be expected by chance. For example, two words *bath* and *towel* cooccur in a text more frequently than would be expected by the frequency of these words because “*bath towel*” is a meaningful phrase used to refer to the towel used after taking a bath. Hence, *bath* and *towel* are syntagmatically related and people often associate *bath* with *towel*. Syntagmatically related words tend to cooccur in a noun phrase (e.g., *red rose*) or a verb phrase (*play soccer*), and thus they are likely to belong to different word classes (e.g., noun–adjective, noun–verb). In other words, syntagmatically related words are not semantically similar, but semantically related. On the other hand, two words are paradigmatically related if they do not cooccur but can be substituted for one another; in other words, they cooccur with similar words. For example, two words *student* and *pupil* are unlikely to cooccur, but they occur in the same context such as “A teacher scolded his [student/pupil] in the classroom,” and thus they are paradigmatically related. Paradigmatic relations tend to be taxonomically or semantically similar by virtue of synonym, antonym or other coordinates, and they belong to the same word class (e.g., noun–noun, verb–verb).

The syntagmatic-paradigmatic distinction, by its definition, is closely related to the way in which contexts are given to construct the initial matrix in the DSM framework [56, 57]. Syntagmatic relations are likely to be represented by the use of documents as contexts, in other words by DSMs with the word-document matrix, because two word vectors (i.e., row vectors of the matrix) are more similar if they cooccur in more documents. Paradigmatic relations are likely to be represented by the use of words as contexts, in other words by DSMs with the word-word matrix, because two word vectors are more similar if they share more collocated words. For example, Table 5 lists the associates (i.e., out-degree neighbors) of the cue words *lemon* and *violet* in the DSM networks based on the initial unsmoothed word-document and word-word matrices, together with the associates in the USF association network. As predicted, more associates in the word-word-based network are paradigmatically related to the cue words than those in the word-document-based network.

**Table 5:** List of associates of some cue words with their semantic relations.

Cue	Network	Associate words (Out-degree neighbors)	# of paradigmatic relation
lemon	DSM (word-document)	juice, pepper, parsley, grate, sauce, butter, seasoning, onion, chicken, mayonnaise, cook	0
	DSM (word-word)	**orange**, carton, **lime**, seasoning, orange juice, **fruit**, zest, juice, mayonnaise, tomato, vinegar	3
	USF association	**lime**, sour, **orange**, **tree**, **fruit**, yellow, car, lemonade, peel, **apple**, squeeze	5
violet	DSM (word-document)	**yellow**, bloom, flower, summer, **blue**, **green**	3
	DSM (word-word)	**pink**, pale, **purple**, **yellow**, **green**, **red**	5
	USF association	**purple**, flower, rose, **color**, **blue**, fem	3

*Note.* Bolded words are paradigmatically related to their corresponding cue word. Both DSM networks used in this table are generated from the unsmoothed and unweighted matrix.

In the next subsection, we explain the results of the hierarchical property for DSM networks obtained in the last section, using the relationship between the syntagmatic-paradigmatic distinction and the DSM matrix.

### Hierarchical Structure of Semantic Networks and Syntagmatic-Paradigmatic Distinction

**Fig 9:**
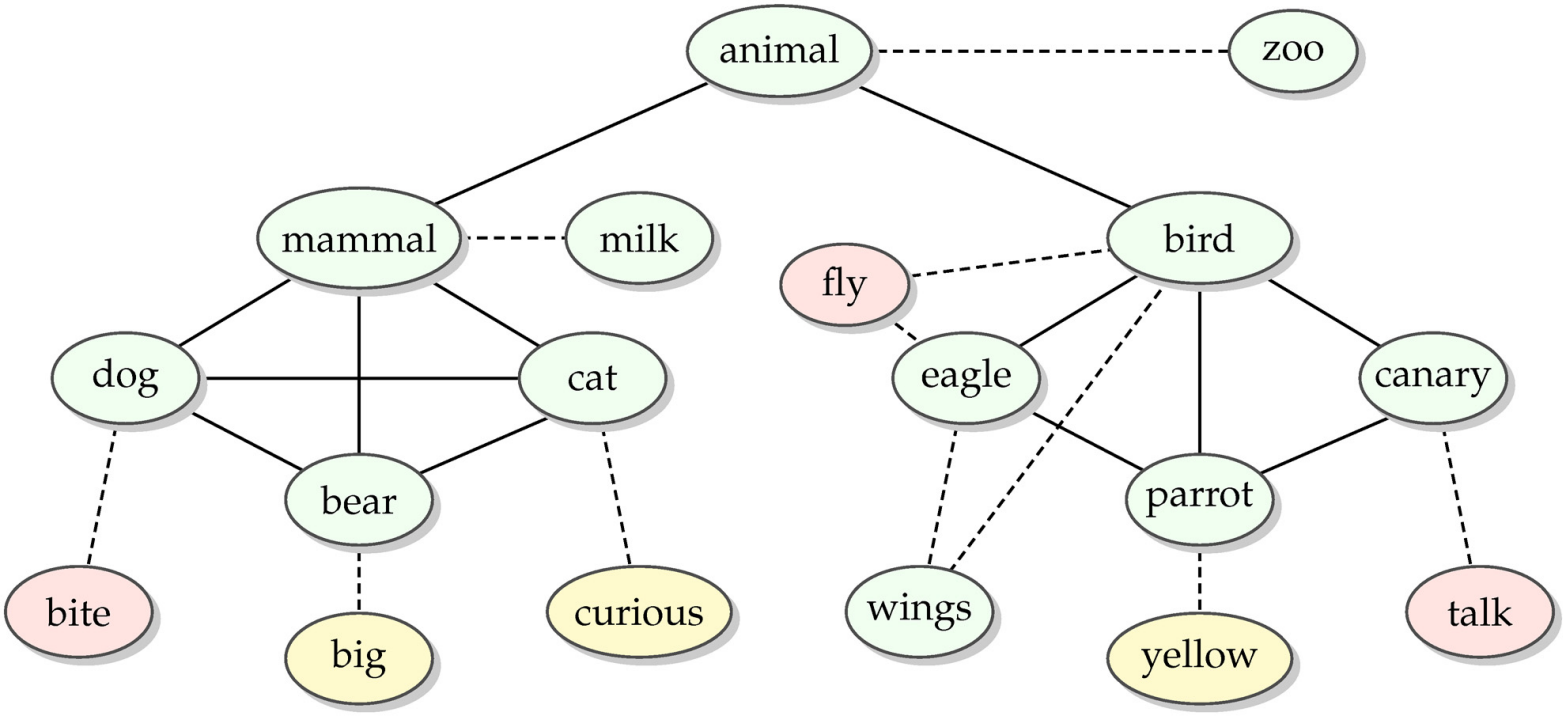
Illustration of the hypothetical semantic network created by syntagmatic and paradigmatic relations. Solid lines denote paradigmatic relations, while dashed lines denote syntagmatic relations. The color of a node indicates the word class of the corresponding word (green = noun, yellow = adjective, red = verb).

Syntagmatic and paradigmatic relations play different roles in creating the hierarchical structure of a semantic network. As explained in the previous subsection, paradigmatically related words are taxonomically similar; they are synonyms or coordinated words (i.e., they share a superordinate word), or one word is a hyponym (or superordinate) of another word. Therefore, a semantic network (a word web) created using paradigmatic relations forms a hierarchy of words or their concepts. In cognitive science, this kind of word hierarchy has been proposed for quite some time as a mental representation of semantic memory, such as Collins and Quillian’s hierarchical network model [1]. A thesaurus (e.g., Roget’s thesaurus and WordNet [58]) is also a typical example of this kind of word hierarchy. In contrast, syntagmatically related words are not semantically (or taxonomically) similar; they are related by virtue of a relation between a concept and its feature or attribute. For example, “being used after a *bath*” is a feature of a *towel* and “being *red*” is a feature of a *rose*. Syntagmatic relations themselves do not form a hierarchical network because they simply join a concept and its features. Psychological models for semantic memory built primarily on the basis of syntagmatic relations do not have a hierarchical structure. For example, feature models [3, 4] argue that a concept (i.e., the meaning of a noun) is represented as an unstructured set of features. Hence, syntagmatic relations are likely to make a semantic network less hierarchical. Fig 9 shows an example of how a semantic network is organized hierarchically by paradigmatic relations and non-hierarchically by syntagmatic relations. In this figure, a hierarchy such as “animal ← mammal ← dog, cat, bear” is formed by paradigmatic relations (denoted by solid lines), while syntagmatic relations (denoted by dashed lines) are not involved in these hierarchical networks.

Considering the relationship of the syntagmatic-paradigmatic distinction to the hierarchical structure of a semantic network and the method used to construct the initial DSM matrix, we can provide a plausible explanation for the results of the hierarchical property of DSM networks. First, DSM networks generated from the unsmoothed word-document matrix are organized non-hierarchically or less hierarchically, because the word-document matrix is likely to represent syntagmatic relations, which lead to a less hierarchical network. Second, DSM networks generated from the unsmoothed word-word matrix are organized hierarchically, because the word-word matrix is likely to represent paradigmatic relations, which organize a hierarchical network. Finally, SVD smoothing induces a change in the hierarchy of semantic networks, because it contributes to the discovery of latent meanings. Latent meanings are not explicitly represented by a word-context matrix, and it follows that the smoothed matrix has a greater chance of capturing semantic relations other than those favored by the original matrix. Therefore, non-hierarchical DSM networks for the word-document matrix become hierarchical, whereas hierarchical DSM networks for the word-word matrix become less hierarchical when the matrices are smoothed.

To confirm that the syntagmatic-paradigmatic distinction really affects the hierarchical structure in semantic networks, we examined the correlation between the degree of network hierarchy and the proportion of edges representing syntagmatic relations. Although there is no widely accepted method to measure the degree of network hierarchy quantitatively, we used a dependency between *C*(*k*) and *k*, namely a correlation coefficient between *C*(*k*) and *k* on a log-log plot. To estimate the proportion of edges connecting syntagmatically related word nodes, we judged whether each edge was based on a syntagmatic or paradigmatic relation according to two criteria based on WordNet [58]: specific patterns of the subgraph (i.e., network motif) connecting two word nodes and a thesaurus-based similarity between these nodes. Because these criteria are conditions for a paradigmatic relation, a pair of words connected by an edge was judged to be syntagmatically related if neither criterion was satisfied. For example, two words were deemed paradigmatically related if they share a parent node in the WordNet hierarchy (i.e., they are synonyms). Two words were also deemed paradigmatically related if one word node is a child or a grandchild of another word node (superordination), or if the two words do not share a parent node but are very closely positioned in the WordNet hierarchy (coordination). Next, for all 24 DSM networks plus the USF association network, we calculated the correlation coefficient between the correlation coefficient between *C*(*k*) and *k* and the proportion of syntagmatically motivated edges. If our assumption is correct, the proportion of edges connecting syntagmatically related words should be positively correlated with the correlation coefficient between *C*(*k*) and *k*, because more syntagmatic relations make a semantic network less hierarchical, and less hierarchical networks are assumed to exhibit a weaker negative correlation (and thus a greater negative value of the correlation coefficient) between *C*(*k*) and *k*.

This correlation analysis revealed that there is a significant positive correlation between the proportion of syntagmatically motivated edges and the correlation coefficient between *C*(*k*) and *k*, *r* = 0.482 (*n* = 25), *p* < .05. This result clearly indicates that the difference in semantic relations does affect the hierarchical structure of a semantic network; semantic networks in which more paradigmatically related words are connected are likely to exhibit a hierarchical structure.

### Network Model and Semantic Development

In this section, we also demonstrate that the syntagmatic-paradigmatic distinction can explain a variety of power-law or truncated power-law distributions revealed by different DSM networks, by incorporating the syntagmatic-paradigmatic distinction into a growing network model for simulating a semantic network.

#### Steyvers–Tenenbaum model

Barabási and Albert [40] proposed a simple model for a scale-free network using the mechanism of network growth and *preferential attachment*, which leads to the pure power-law degree distribution with an exponent of 3. In this model, a small fully connected network of *M*_0_ nodes is initially constructed, and then new nodes are successively added to the network (i.e., network growth). Each new node is connected to *M*(≤ *M*_0_) existing nodes selected with probability proportional to their degrees (i.e., preferential attachment).

To provide a psychologically plausible explanation of semantic growth, Steyvers and Tenenbaum [21] extended the Barabási–Albert model by introducing the process of *semantic differentiation* into the simple mechanism of preferential attachment. They assumed that semantic differentiation is the process of adding some kind of variation on the meaning of an existing word by a new word, according to the suggestions made by studies on language and conceptual development. Their network model differentiates the meaning of an existing word node by connecting a new node to *M* randomly chosen neighbors of the existing node. Steyvers and Tenenbaum [21] demonstrated that the degree distribution of the network generated by this model fits well with the observed distribution of the word association network. However, their model cannot explain the observed difference among distributions of DSM networks, because it does not have sufficient free scaling parameters to generate a variety of pure and truncated power-law distributions.

It should be noted that Steyvers and Tenenbaum [21] provided a more general formalization of the probability *P*_*ij*_ with which a particular node *v*_*j*_ is chosen in the neighborhood of *v*_*i*_ at Step 2 as given below:

$$P_{ij}=\frac{u_{j}}{\sum_{v_{k}\in H_{i}}u_{k}}$$ (6)

where *u*_*j*_ is the utility of a word of *v*_*j*_, and *H*_*i*_ is a set of neighbors of *v*_*i*_. They suggested two ways of determining node utilities: utility based on word frequency (*u*_*j*_ = log(*f*_*j*_ + 1) where *f*_*j*_ is the frequency of a word *v*_*j*_) and an equal utility (*u*_*j*_ = 1/|*H*_*i*_|). They reported that these two utility functions yielded very similar results for degree distributions. Additionally, although they did not describe how the frequency of words in real semantic networks was used to determine the utility of nodes in a synthetic network, it is very difficult to determine how this should be done because the order of adding words with different frequencies to the existing network affects the behavior of the resulting synthetic network. For these reasons, in this study we used the equal utility function, thereby assuming that the probability of choosing a neighbor node is uniformly distributed, as described in the algorithm above.

#### New network model

We argue that the limitation of the Steyvers–Tenenbaum model can be overcome by assuming that semantic growth cannot be explained solely by the process of semantic differentiation. Obviously, two word nodes connected by semantic differentiation (or preferential attachment) can be regarded as semantically or taxonomically similar (i.e., paradigmatically related), because a new word added to the network by semantic differentiation corresponds to more specific variations on existing words. However, as explained thus far, a new word can be associated with other words by a syntagmatic relation. Furthermore, this process of acquiring the connections by syntagmatic relations does not necessarily require preferential attachment, because there is no reason to assume that highly complex concepts (i.e., those with many connections) are likely to be attributes of a new concept. For example, in the USF association norms, the four most listed associates of the cue word *cherry* are *red*, *pie*, *fruit*, and *apple*. The words *fruit* and *apple* are paradigmatically related to *cherry*, while *red* and *pie* are syntagmatically related to *cherry*. When we consider the situation where a new word *cherry* is added to the network, adding edges from *cherry* to *fruit* and *apple* means that *cherry* differentiates the concepts of *fruit* and *apple* by introducing their new subcategories. This acquisition process can be reasonably regarded as semantic differentiation implemented by preferential attachment. On the other hand, adding edges to *red* and *pie* is interpreted differently; these edges may be added because *cherry* has the attributes of “being red” and “being an ingredient of a pie”. In this case, it is not reasonable to assume that preferential attachment always governs this process. These edges are added simply because *cherry* has the attributes of “being red” and “being an ingredient of a pie,” and not because *red* and *pie* have a variety or wide range of meanings to be differentiated. Hence, the acquisition process based on syntagmatic relations should be modeled by a different mechanism other than semantic differentiation. We refer to this mechanism as *experiential correlation*, which is a term borrowed from cognitive linguistics that indicates perceptual correspondences grounded in human embodied experience [59].

To integrate the process of experiential correlation into the Steyvers–Tenenbaum model, we consider *random attachment* of a new node to the existing nodes. As mentioned above, there is no reason to assume that highly complex concepts with many connections are likely to be attributes of a new concept. It can be justified by empirical observation that verb and adjective concepts involve much less hierarchical complexity than noun concepts; verb and adjective concepts are represented by fewer levels of hierarchy (generally two) and with fewer distinctions at the superordinate level than noun concepts [60]. Furthermore, because it is very difficult to assume a priori information on the attribute of a new concept (e.g., which color is preferentially selected as an attribute of a new object), we apply the principle of indifference to the process of experiential correlation by assuming that the probability of choosing an existing node for experiential correlation is equal over all existing nodes. It should be noted, however, that we do not claim that experiential correlation is never governed by preferential attachment or other network growth processes. We simply argue that random attachment is a plausible assumption when no a priori knowledge is available for node choice for experiential correlation. In some cases, preferential attachment may work as a principle of experiential correlation. For example, some adjectives have a more basic, wider meaning than other relevant adjectives (e.g., *red* versus *reddish*).

Liu et al. [61] integrated random attachment into the Barabási–Albert model. In their model, a new node is attached to the existing nodes preferentially with probability 1 − *p* or randomly with probability *p*. The resulting network has a degree distribution that follows a mixture of power-law and exponential behaviors. Clearly, the distribution completely follows the power law if *p* = 0, whereas it follows the exponential if *p* = 1. When 0 < *p* < 1, the distribution exhibits an approximately exponential behavior for small *k*, and a power-law-like behavior for large *k*. Note that, as mentioned earlier, the degree distribution of the USF association network follows the exponential below *k*_*min*_ and the truncated power law above *k*_*min*_. This suggests that both preferential and random attachments may be required for appropriately simulating the behavior of semantic networks.

Following Liu et al.’s model [61], we propose a new network growth model by extending the Steyvers–Tenenbaum model to enable both preferential and random attachments. A new node is attached to the existing network preferentially by semantic differentiation with probability 1 − *p*, and randomly by experiential correlation with probability *p*. For preferential attachment, nodes connected to a new node are chosen from among only the neighbors of node *v*_*i*_ that were previously added by preferential attachment. In random attachment, nodes are chosen from among all the existing nodes.

A more formal description of the algorithm for connecting a new node to *M* existing nodes in our modified Steyvers–Tenenbaum model is given below.

1. Each of the *M* edges connecting a new node to the existing network is labeled as either “semantic differentiation” with probability 1 − *p* or “experiential correlation” with probability *p*. The resulting numbers of edges labeled as “semantic differentiation” and “experiential correlation” are denoted by *M*_*p*_ and *M*_*r*_(= *M* − *M*_*p*_), respectively.
2. *M*_*p*_ existing nodes are chosen and connected to a new node as follows. According to the original Steyvers–Tenenbaum model, an existing node *v*_*i*_ is chosen for differentiation with probability proportional to its degree. *M*_*p*_ nodes are chosen randomly from the neighbors of node *v*_*i*_ labeled as “semantic differentiation.”
3. *M*_*r*_ existing nodes are chosen randomly (i.e., with equal probability) from all existing nodes, and connected to the new node.
4. The direction of each edge is determined randomly, pointing toward the existing node with probability *γ* and toward the new node with probability 1 − *γ*, as in the case in the Steyvers–Tenenbaum model.

#### Simulation results

We conducted a simulation experiment modeling the behavior of the USF and DSM networks using the modified Steyvers–Tenenbaum model. As the target for simulation, we considered three representative DSM networks created by the *cs*-method; these were generated from the tf-idf-weighted and smoothed word-document matrix (corresponding to LSA), the unweighted and unsmoothed word-word matrix, and the ppmi-weighted and smoothed word-word matrix, respectively. These target networks were chosen such that different types of degree distributions (i.e., Type M, Type L, and Type S) were simulated. As shown in Table 4, the USF association network is of Type M, the two DSM networks generated from the tf-idf-weighted and smoothed word-document matrix and the ppmi-weighted and smoothed word-word matrix are of Type S, and the DSM network generated from the unweighted and unsmoothed word-word matrix is of Type L. DSM networks of Type F were not used in the simulation, because they deviate from naturally occurring semantic networks owing to the unsuccessful construction of word-document-based networks as explained previously. In all the simulations, we set *n* equal to the number of nodes in the real networks (i.e., *n* = 5,018 for the USF association network and *n* = 4,702 for the DSM networks). We also set *M* = 13 to ensure that the resulting synthetic networks would have approximately the same density as the corresponding real networks.

Two important parameters for the model, *p* and *γ*, were set as follows. We determined *p* using the fraction *q* of edges connecting a pair of syntagmatically related words in the target network and compared three settings: *p* = *q* (Model A), *p* = *q*/2 (Model B) and *p* = 0 (Model ST). Model A assumes that all syntagmatic relations are the result of random attachment, while Model B assumes that syntagmatic relations are caused equally by random and preferential attachment. We also consider Model ST, corresponding to the original Steyvers–Tenenbaum model, to compare the simulation performance of the modified Steyvers–Tenenbaum model and the original one. Note that the rationale behind Model B is that some syntagmatic relations may be governed by preferential attachment. As mentioned earlier, some verbs or adjectives are more general than others (e.g., *red* versus *reddish*). Some syntagmatically related nouns can also be assumed to be connected by means of semantic differentiation. For example, *cherry* and *pie* are syntagmatically related because *cherry* has the attribute of “being an ingredient of a pie,” but we also assume that *cherry* subcategorizes a concept *pie* (i.e., a cherry pie is a specific kind of pie). Because we have no prior knowledge of how likely syntagmatic relations are to be caused by preferential attachment, we simply assume equal probability for preferential and random attachment for syntagmatically related nodes.

Parameter *γ* was determined such that the resulting synthetic network would have approximately the same connectivity *n*_*CC*_ as the corresponding real network. Parameter *γ* is generally correlated with *n*_*CC*_; a very high value of *γ* close to 1 generates a network with low *n*_*CC*_, and *n*_*CC*_ increases as *γ* decreases. Therefore, we decreased *γ* from 1 in steps of 0.005 (or 0.001 for a very low values of *n*_*CC*_ of a real network), and generated 50 networks for each *γ* value. We then calculated the average *n*_*CC*_ over the 50 networks and determined *γ* to be the value that achieved the closest *n*_*CC*_ to the real network. This parameter tuning process was conducted for each of the three models simulating a real network. To analyze the model’s performance, of the 50 synthetic networks, we chose one whose *n*_*CC*_ was closest to that of the corresponding real network. All the results reported below were obtained for the chosen network.

Table 6 summarizes the statistics of the synthetic networks generated by the model together with those of the corresponding real networks. Table 6 demonstrates that all the synthetic networks have the small-world property; the average shortest path length *L* and diameter *D* are close to those of the real network, and the clustering coefficient *C* is higher than that of the random network. One difference is that the clustering coefficient *C* of Model A is relatively low compared with the real network. This result is not surprising because the higher probability *p* of random attachment generates a more randomized, and thus less clustered network.

**Table 6:** Statistics for the simulated network generated by the proposed network models.

	*m*	*n*_*CC*_	⟨*k*⟩	*D*	*L*	*C*	*D*_*KS*_	*r*	*β*	*L*_*random*_	*C*_*random*_
USF association (Type M; *n* = 5,018)											
Model A	65,221	4,845	13.0	10	4.07	0.050	0.068	−.069	0.018	—	—
Model B	65,221	4,844	13.0	10	3.92	0.147	0.050	−.488	0.517	—	—
Model ST	65,221	4,845	13.0	9	3.80	0.261	0.076	−.708	0.698	—	—
Data	63,620	4,845	12.7	10	4.26	0.187	—	−.706	0.750	3.64	0.005
Word-word matrix, unweighted, unsmoothed, *cs*-method (Type L; *n* = 4,702)											
Model A	61,113	3,097	13.0	26	6.49	0.027	0.121	.090	-0.346	—	—
Model B	61,113	3,099	13.0	28	6.19	0.137	0.077	−.393	0.431	—	—
Model ST	61,113	3,091	13.0	19	5.52	0.266	0.056	−.712	0.661	—	—
Data	59,621	3,091	12.8	24	8.31	0.366	—	−.730	0.644	3.45	0.008
Word-document matrix, tf-idf, smoothed, *cs*-method (Type S; *n* = 4,702)											
Model A	61,113	4,157	13.0	16	4.76	0.025	0.050	.059	-0.535	—	—
Model B	61,113	4,158	13.0	12	4.50	0.129	0.081	−.399	0.424	—	—
Model ST	61,113	4,158	13.0	10	4.25	0.270	0.119	−.715	0.703	—	—
Data	59,613	4,156	12.6	27	5.83	0.317	—	−.487	0.482	3.58	0.006
Word-word matrix, ppmi, smoothed, *cs*-method (Type S; *n* = 4,702)											
Model A	61,113	4,474	13.0	10	4.20	0.041	0.096	−.022	-0.135	—	—
Model B	61,113	4,474	13.0	9	4.04	0.141	0.128	−.482	0.492	—	—
Model ST	61,113	4,474	13.0	8	3.89	0.266	0.147	−.708	0.704	—	—
Data	59,613	4,474	12.7	19	5.77	0.251	—	−.383	0.477	3.60	0.006

*Note.* *n* = number of nodes; *m* = number of edges; *n*_*CC*_ = number of nodes of the largest (strongly) connected component; ⟨*k*⟩ = average node degree; *D* = diameter of the network; *L* = average shortest path length; *C* = clustering coefficient; *D*_*KS*_ = Kolmogorov–Smirnov statistic between the model and the data; *r* = correlation coefficient between local clustering coefficient *C*(*k*) and node degree *k* on a log-log plot; *β* = hierarchical exponent; *L*_*random*_ = average shortest path length of the random network with the same size and density; *C*_*random*_ = clustering coefficient of the random network with the same size and density.

**Fig 10:**
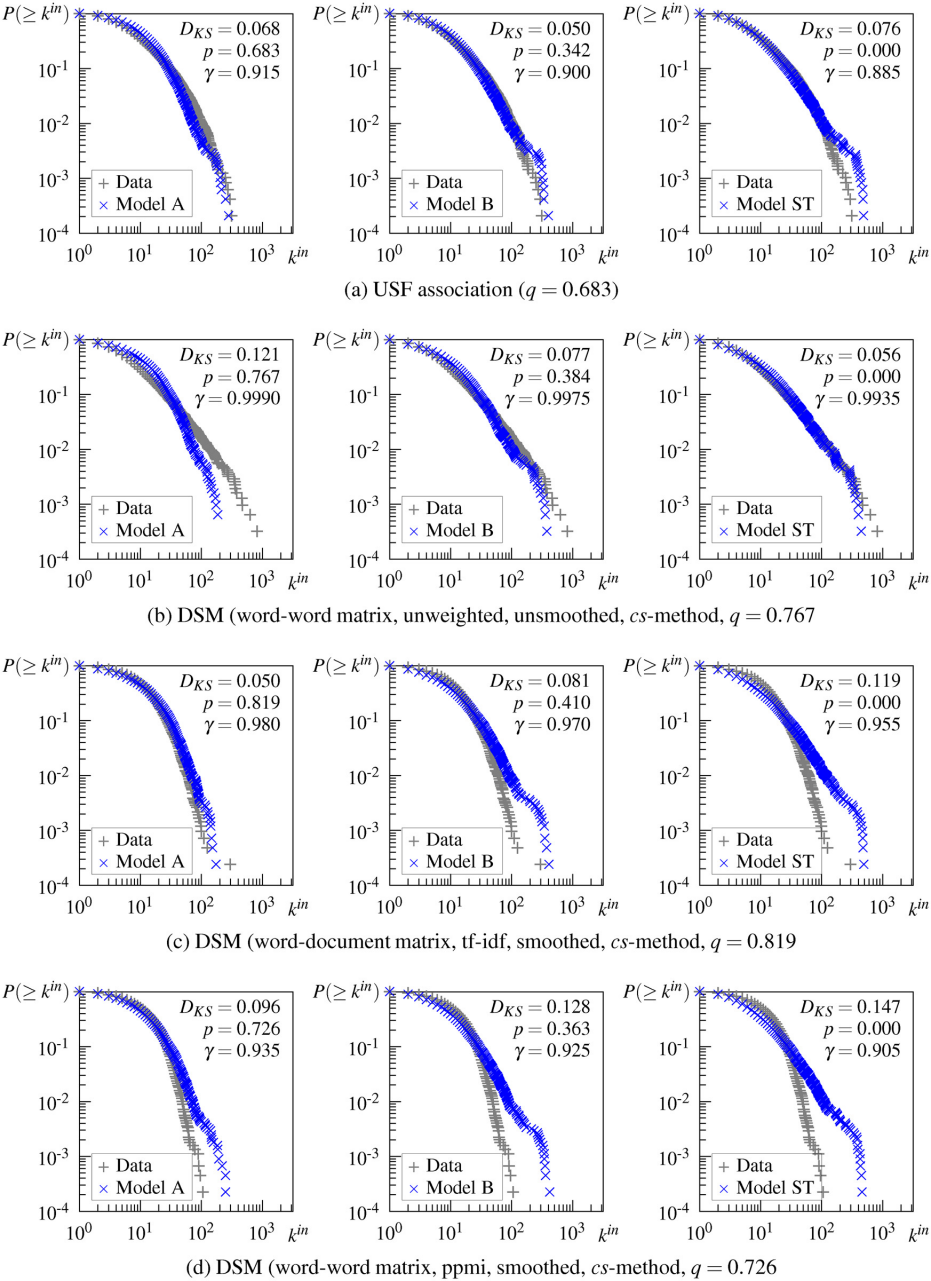
Cumulative in-degree distributions of the synthetic networks generated by the modified Steyvers–Tenenbaum model (Model A and Model B) and by the original Steyvers–Tenenbaum model (Model ST). Note that *D*_*KS*_ denotes the Kolmogorov–Smirnov statistic, *p* denotes the probability of random attachment, and *γ* is the probability of a new node pointing toward the existing node.

Fig 10 shows the cumulative in-degree distributions of the synthetic networks generated by the three models (Model A, Model B and Model ST). To quantitatively evaluate how well the distributions of the model fit those of the real networks, we also show the Kolmogorov–Smirnov statistic *D*_*KS*_ in the graphs and in Table 6. A smaller *D*_*KS*_ implies a better fit between the model and the data. In Fig 10, the best-fit model differs depending on the real network simulated. Model A reproduces the in-degree distribution that best fits the data for the DSM networks of Type S generated by smoothed matrices, as shown in Fig 10c and 10d. Model B is most appropriate for simulating the distribution of the USF association network (Type M), as shown in Fig 10a. Model ST also generates an appropriate distribution in some cases; it best fits the DSM network of the unsmoothed word-word matrix (Type L), as shown in Fig 10b. These results can be summarized as the relationship between the overall shape (or the tail behavior) of the in-degree distribution and the probability *p* of random attachment. A network model with a higher *p* (Model A > Model B > Model ST) produces a more truncated distribution (Type S > Type M > Type L).

This difference of the best-fit model suggests that how likely semantically related words are to be connected by random attachment cannot be explained solely by the syntagmatic-paradigmatic distinction. Although we do not provide a reasonable explanation for why this difference occurs as this requires further research, these results imply that the ability of the original Steyvers–Tenenbaum model is limited. A network model with both preferential and random attachments is generally more appropriate to explain the in-degree distribution of semantic networks (especially, the real association network) than the original Steyvers–Tenenbaum model.

**Fig 11:**
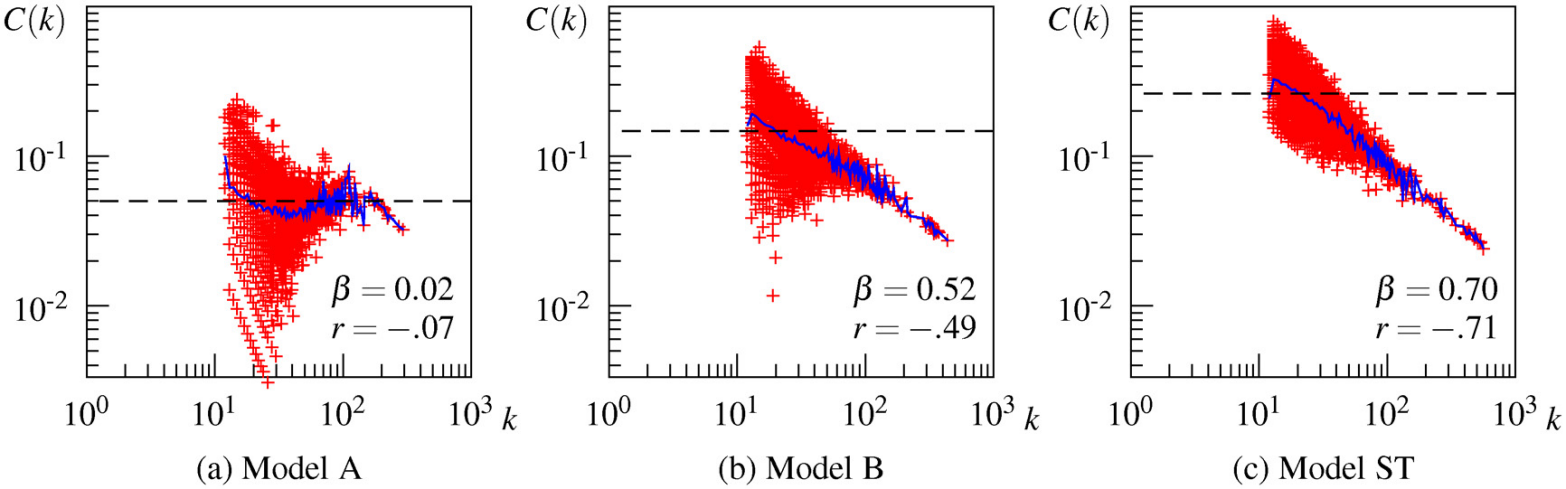
Local clustering coefficient as a function of the node degree for three synthetic networks generated by the modified Steyvers–Tenenbaum model (Model A and Model B) and by the original Steyvers–Tenenbaum model (Model ST): case of simulating the USF association network. Red plots denote the local clustering coefficient of an individual node, the blue line connects the average local clustering coefficient with the same degree, and the dashed line denotes the clustering coefficient *C*.

Regarding the hierarchical structure of the synthetic networks, the scaling behavior of the local clustering coefficient *C*(*k*) of the synthetic networks is shown in Fig 11. Table 6 also lists the correlation coefficient *r* between *C*(*k*) and *k*, and the hierarchical exponent *β*. Fig 11 only shows the results for three synthetic networks generated to simulate the data of the USF association network; however the results for the DSM networks do not differ from these ones. Overall, the probability *p* determines the distribution of local clustering coefficients. A model with a lower value of *p* yields a stronger negative correlation between *C*(*k*) and *k* and a higher *β*, and thus creates a more hierarchically structured network. The hierarchical modularity of the USF association network (Fig 2) and the unsmoothed word-word-based DSM network (Fig 8d) is reproduced best by Model ST, while the hierarchical modularity of the smoothed DSM networks (Fig 8c–8e) is most similar to that of Model B. This result is not unexpected, because random graph models cannot generate a structured network in which *C*(*k*) correlates with the degree *k* [46]. Hence, a network in which more edges are generated by random attachment (i.e., generated by models with higher *p*) tends to be less hierarchically structured.

The simulation results reported are summarized in Table 7. The scale-free property of the USF network and many DSM networks can be explained more plausibly by the modified Steyvers–Tenenbaum model (Model A and B), although whether Model A or B is a better fit for the observed distribution depends on semantic networks. On the other hand, the hierarchical property of the semantic networks is captured better by the original Steyvers–Tenenbaum model or the modified model (Model B). Taken together, in many cases the proposed model better explains the real semantic networks, and Model B seems to provide a balanced account of both network properties.

**Table 7:** Summary of simulation results: The best model for simulating the network properties of each real semantic network.

Network (Type of degree distribution)	Network property		
	Small-world	Scale-free	Hierarchical
USF association (Type M)	All models	Model B	Model ST
Word-word matrix, unweighted, unsmoothed (Type L)	All models	Model ST	Model ST
Word-document matrix, tf-idf, smoothed (Type S)	All models	Model A	Model B
Word-word matrix, ppmi, smoothed (Type S)	All models	Model A	Model B

The proposed network model has several limitations. In this paper, we aim to demonstrate the validity and merits of simple integration of random attachment into the network model for lexical development, rather than providing a network model that can simulate the real semantic networks as accurately as possible. The most serious limitation of the proposed model is that, as shown in Table 7, the optimal value of *p* (i.e., the probability of random attachment) in the proposed model differs between the degree distribution and the hierarchical structure; the degree distribution of a real network is appropriately explained by models with high values of *p* (i.e., Model A or B), whereas models with lower values of *p* (i.e., Model B or ST) than these ones are more appropriate for the highly hierarchical structure of the same real network. These incompatible results are attributed to the limitation of the simple idea of regarding random attachment as a model of experiential correlation. To build a model that can consistently explain both the degree distribution and hierarchical structure, we need to explore how human lexical knowledge is acquired through experiential correlation and integrate such a mechanism into the proposed network model.

Although a more plausible model requires further research, we suggest some possible modifications that would achieve more precise simulation of the behavior of real semantic networks. One possible modification relates to the utility of neighbors (Eq 6) being connected to a new node in the process of semantic differentiation. If the choice of neighbors is governed by the idea of preferential attachment, for example, based on word frequency or other properties, the modified Steyvers–Tenenbaum model may be able to simulate the hierarchical structure more precisely. As a related modification, we can also assume a model that connects a new node to the node chosen for semantic differentiation as well as *M* − 1, instead of *M*, neighbors of the chosen node. Additionally, to reproduce a more realistic degree distribution and scaling of local clustering coefficients *C*(*k*), we can modify the model such that the number of edges *M* added to the network fluctuates. For example, use of the Poisson distribution with a mean equal to ⟨*k*⟩ of a real semantic network may be able to simulate an in-degree distribution and a *C*(*k*) distribution for low values of *k* more precisely. Finally, we may need to develop a more plausible model of experiential correlation by considering the characteristics of syntagmatically related words (i.e., difference among word classes) to simulate the in-degree distribution more precisely. It is worth pursuing these extensions in future research.

## Discussion

The complex network analysis reported in this paper demonstrates that all the DSM networks have the same small-world property as the association network. Furthermore, some DSM networks (especially, DSM networks generated from the weighted and unsmoothed word-word matrices) have the same scale-free and hierarchical properties as the association network. From this result we can conclude that DSM provides a plausible model of human semantic memory; this is in contrast to Steyvers and Tenenbaum’s [21] argument that LSA and other semantic spaces are limited as a model of human semantic memory. They argued that the degree distribution of the USF association network follows a pure power law, whereas the distribution of the LSA network does not. However, our analyses and other similar analyses [29] yield a different result. The degree distribution of the USF association network follows not a pure power law, but a truncated power law with an initially exponential decay. Indeed, a closer look at Steyvers and Tenenbaum’s [21] plotted distribution reveals that it deviates from a pure power law. This difference in the scale-free structure of the association network may be the primary reason for the incompatibility between their arguments and ours regarding the ability of the DSM. Furthermore, in our analysis, the degree distribution of the LSA network (which corresponds to the DSM network created from the tf-idf-weighted and smoothed word-document matrix) also exhibits a similar degree distribution, although its power-law slope is steeper than that of the association network. More importantly, some semantic spaces generated by DSM methods other than LSA (e.g., semantic spaces generated from the weighted and unsmoothed word-word matrices) yield a degree distribution very similar to that of the association network. Steyvers and Tenenbaum [21] analyzed only the semantic network based on LSA, and speculated that other semantic spaces are unlikely to reproduce scale-free connectivity. This speculation is derived from the assumption that LSA and other semantic spaces share geometric properties of Euclidean-space semantic representations that are not consistent with human similarity judgments. Our findings indicate that this speculation is not valid. Hence, it provides empirical evidence of the DSM’s ability to simulate the network structure underlying human semantic knowledge or word association; this is one of the original contributions of this study.

Regarding the psychological plausibility of the DSM as a model of human semantic knowledge, a number of existing studies [33, 35, 62, 63] have already provided empirical evidence by demonstrating that the DSM well explains human performance on behavioral tasks (e.g., similarity judgment and semantic priming) for some individual words. These findings demonstrate the plausibility of DSMs by focusing on the microscopic behavior that emerges from semantic knowledge, but they divulge little about the holistic structure. In contrast, our analysis reveals that the DSM can generate semantic spaces whose holistic structure is similar to that of human semantic knowledge; thus, this study provides new evidence for the argument that the DSM is a plausible model of semantic memory.

The findings on network properties of different DSM networks have several implications for the theory of DSMs. First, the DSM networks constructed from the weighted and unsmoothed word-word matrix best simulate all the network properties of the USF association network; these DSM networks are small-world (as shown in Table 2), follow a truncated power law almost identical to the distribution of the association network (as shown in Fig 6b–6d), and have the same degree of hierarchical structure as the association network (as shown in Fig 8e). This finding indicates the advantage of word cooccurrence statistics, or a words-as-contexts method, for modeling human semantic knowledge. Some previous studies [34, 64] have demonstrated that DSMs based on word cooccurrence statistics achieve higher performance on lexical tasks such as similarity judgment and synonym identification, but it is not clear whether this superiority holds for the modeling accuracy of the structure of semantic knowledge or the mental lexicon. The network analysis presented in this paper is a first step toward identifying theoretical evidence for the DSM’s ability to model the holistic structure of semantic knowledge, and it demonstrates that DSMs based on word cooccurrence have this ability.

Second, the observed differences in network properties between the word-word- and word-document-matrix-based DSMs provide empirical evidence of the effect of the initial matrix’s context type on the semantic properties of constructed semantic spaces [56, 57]. As described earlier, Sahlgren [56] argued that a word-document matrix is likely to assign higher similarity to syntagmatically related words, while a word-word matrix is likely to assign higher similarity to paradigmatically related words. Utsumi [57] justified his argument by comparing the performance of the two types of matrices in predicting word association. The network analysis presented in this paper provides new empirical evidence for his argument from a different perspective. Hierarchical structure in DSM networks, which is assessed by a power-law relationship between node degree *k* and local clustering coefficient *C*(*k*), is explained in accordance with the argument for the relationship between DSM matrices and semantic relations.

Finally, SVD smoothing does not improve the performance of simulating the network properties of a real association network as well as generally expected in the DSM framework. Smoothing yields a considerable effect on DSM networks based on the word-document matrix, but the obtained scale-free and hierarchical properties are not sufficiently consistent with those of the association network. For the word-word matrix, smoothed DSMs exhibit network properties slightly less consistent with the association network than unsmoothed DSMs. In particular, when a word-word matrix is weighted by tf-idf or ppmi, SVD smoothing results in a more truncated degree distribution and a less hierarchical structure than the association network. This result seems to show that SVD smoothing is not effective in developing a cognitive model of human semantic knowledge or the mental lexicon; in other words, excessive discovery of latent meanings may have a harmful effect on modeling human semantic knowledge. It may suggest that latent meanings revealed by SVD smoothing are not explicitly represented in human semantic knowledge, or at least they do not emerge in human behavior of free word association. However, a more reasonable explanation of this result could be that SVD smoothing of a word-word matrix generally fails to improve the performance of similarity judgment. The semantic information involved in a (ppmi-weighted) word-word matrix is richer than a word-document matrix and becomes even richer as the corpus size increases. A recent study [65] reported a finding supporting this assumption. The authors demonstrated that SVD smoothing of a ppmi-weighted word-word matrix does not improve the performance on semantic tasks such as a multiple-choice synonym test and semantic categorization. Note that this study also reported an interesting finding that dimensionality reduction using SVD achieved significant improvements in all semantic tasks when the contributions of the initial dimensions (corresponding to large singular values) were reduced, rather than when all dimensions contributed equally as in the usual manner for DSMs. Therefore, DSMs using this kind of unbalanced smoothing may be effective in modeling semantic knowledge.

The simulation results of network growth demonstrate that, in many cases, the modified Steyvers–Tenenbaum model with both preferential and random attachments better reproduces real semantic networks. This result suggests that preferential attachment alone is insufficient; both preferential and random attachments are necessary to explain the developmental process of semantic or lexical knowledge. Given that preferential attachment or semantic differentiation is basically a process of acquiring unknown words paradigmatically related to already-known words, it also suggests that lexical development is motivated by semantic relations other than paradigmatic or taxonomic ones. We argue that syntagmatic-paradigmatic distinction of semantic relations is critical in explaining this result. Concerning adult semantic knowledge, a large number of lexical priming experiments [66–69] have shown that its structure is underpinned by both paradigmatic (or taxonomic) and syntagmatic (or associative) relations. In these experiments, reliable priming effects are repeatedly observed for taxonomically related pairs (e.g., *cherry—fruits*) and associatively related pairs (e.g., *cherry—soda*), although very often there is a confounding between taxonomic (or semantic) and associative relatedness in the pairs used in the experiments [67]. Furthermore, Arias-Trejo and Plunkett [70] have recently found that 24-month-old infants exhibited a priming effect for both taxonomically related word pairs and associatively related word pairs. To distinguish precisely between the two types of semantic relations, they carefully selected taxonomically related words that are not associatively related, and associatively related words that are not taxonomically related; hence, their finding does indicate that early lexical development is also driven by both taxonomic and associative relations. The findings of this study are interpreted as lending theoretical support to the need for both taxonomic and associative relations to explain lexical development.

## Conclusion

In this paper, we examined the network structure of DSMs using complex network analysis and reported the following findings: (a) Some DSMs have the ability to generate semantic networks with the same scale-free and hierarchical properties as the USF association network, suggesting that the DSM is a plausible model of human semantic memory; (b) Different patterns of observed network properties of DSM networks reflect a way of constructing semantic spaces, suggesting that complex network analysis provides a new method for exploring the properties and structures of different DSMs in a systematic way; (c) Considering both preferential and random attachments as a mechanism for network growth better explains the network structures observed in the association and DSM networks, suggesting that lexical development cannot be explained solely by the process of semantic differentiation for paradigmatically related words. To the best of our knowledge, no previous studies have applied network analysis to various DSMs in a comprehensive way. This is the first study to analyze the network structure of semantic knowledge represented by a DSM, and thus provides an original contribution to research on DSMs.

## Supporting Information

S1 Table Statistics for all 24 distributional semantic model (DSM) networks (*n* = 4,702). (PDF)

S2 Table Detailed results of statistical tests of power-law behavior for the degree distributions of all 24 DSM networks. (PDF)

S1 Fig In-degree and cumulative in-degree distributions of all 24 DSM networks. (a) in-degree distributions of DSM networks generated from the word-document matrix, (b) in-degree distributions of DSM networks generated from the word-word matrix, (c) cumulative in-degree distributions of DSM networks generated from the word-document matrix, (d) cumulative in-degree distributions of DSM networks generated from the word-word matrix.
(PDF)

S2 Fig Local clustering coefficient *C*(*k*) as a function of degree *k* for all 24 DSM networks. (a) local clustering coefficient as a function of the node degree for DSM networks generated from the word-document matrix, and (b) local clustering coefficient as a function of the node degree for DSM networks generated from the word-word matrix. Red plots denote the local clustering coefficient of an individual node, the blue line connects the average of the local clustering coefficient with the same degree, and the dashed line denotes the clustering coefficient *C*.
(PDF)
