# Supplementary material for: A Complex Network Approach to Distributional Semantic Models
Source: PLoS One. 2015 Aug 21;10(8):e0136277. doi: 10.1371/journal.pone.0136277 (PMC4546414; doi:10.1371/journal.pone.0136277)
Supplement: S1 Table — (PDF) [file pone.0136277.s001.pdf]

Table S1. Statistics for all 24 distributional semantic model (DSM) networks ($n = 4,702$).

weighting / smoothing / neighborhood method		m	n_{CC}	$\langle k \rangle$	k_{max}	D	L	L_{random}	C	C_{random}
Word-document matrix										
unweighted / -svd / k-nn	Directed	60262	4519	12.8	1894	13	4.84	3.59	0.222	0.006
	Undirected	49274	4702	21.0	2044	5	2.94	3.06	0.228	0.005
unweighted / -svd / cs	Directed	59613	4172	12.2	1733	19	5.65	3.62	0.242	0.006
	Undirected	52343	4702	22.3	1994	6	3.08	3.00	0.237	0.005
tf-idf / -svd / k-nn	Directed	60252	4403	12.8	2193	14	5.05	3.58	0.257	0.006
	Undirected	50219	4702	21.4	2456	5	2.83	3.04	0.270	0.005
tf-idf / -svd / cs	Directed	59613	4089	12.1	1977	21	5.85	3.62	0.274	0.006
	Undirected	52733	4702	22.4	2352	6	2.96	3.00	0.274	0.005
ppmi / -svd / k-nn	Directed	60250	4471	12.8	1085	15	4.93	3.59	0.219	0.006
	Undirected	49633	4702	21.1	1219	5	3.14	3.05	0.225	0.005
ppmi / -svd / cs	Directed	59613	4201	12.3	1239	24	5.68	3.61	0.241	0.006
	Undirected	52387	4702	22.3	1424	6	3.20	3.00	0.238	0.005
unweighted / +svd / k-nn	Directed	60251	4182	12.9	127	21	5.94	3.55	0.343	0.006
	Undirected	48745	4702	20.7	148	7	3.86	3.07	0.326	0.005
unweighted / +svd / cs	Directed	59613	3897	12.6	176	23	5.92	3.55	0.331	0.006
	Undirected	49925	4702	21.2	201	8	3.86	3.04	0.318	0.005
tf-idf / +svd / k-nn	Directed	60250	4247	12.8	235	20	5.71	3.57	0.331	0.006
	Undirected	47470	4702	20.2	258	7	3.92	3.10	0.322	0.005
tf-idf / +svd / cs	Directed	59613	4156	12.6	296	27	5.83	3.58	0.317	0.006
	Undirected	48622	4702	20.7	321	8	3.88	3.07	0.308	0.005
ppmi / +svd / k-nn	Directed	60251	4217	12.9	79	21	5.75	3.55	0.327	0.006
	Undirected	47827	4702	20.3	93	7	3.92	3.09	0.312	0.005
ppmi / +svd / cs	Directed	59613	4154	12.6	116	33	6.08	3.58	0.313	0.006
	Undirected	48990	4702	20.8	129	8	3.89	3.06	0.299	0.005
Word-word matrix										
unweighted / -svd / k-nn	Directed	60251	3296	13.2	831	24	8.25	3.43	0.363	0.008
	Undirected	55785	4702	23.7	1122	6	3.06	2.95	0.336	0.005
unweighted / -svd / cs	Directed	59621	3091	12.8	821	24	8.31	3.45	0.366	0.008
	Undirected	55520	4702	23.6	1193	7	3.05	2.95	0.335	0.005
tf-idf / -svd / k-nn	Directed	60252	522	14.0	152	21	6.95	2.66	0.417	0.054
	Undirected	59004	4702	25.1	301	7	3.40	2.91	0.234	0.006
tf-idf / -svd / cs	Directed	59613	455	12.0	130	26	7.22	2.74	0.409	0.052
	Undirected	58528	4702	24.9	308	7	3.38	2.92	0.221	0.006
ppmi / -svd / k-nn	Directed	60250	3748	13.1	199	26	6.08	3.50	0.294	0.007
	Undirected	51316	4702	21.8	235	7	3.54	3.02	0.255	0.005
ppmi / -svd / cs	Directed	59613	3679	12.5	215	24	6.21	3.54	0.275	0.007
	Undirected	52359	4702	22.3	252	7	3.49	3.00	0.234	0.005
unweighted / +svd / k-nn	Directed	60251	3752	13.2	366	19	6.37	3.49	0.339	0.007
	Undirected	52881	4702	22.5	428	7	3.48	2.99	0.312	0.005
unweighted / +svd / cs	Directed	59613	3563	12.8	388	18	6.35	3.50	0.342	0.007
	Undirected	52700	4702	22.4	473	7	3.48	3.00	0.308	0.005

weighting / smoothing / neighborhood method		m	n_{CC}	$\langle k \rangle$	k_{max}	D	L	L_{random}	C	C_{random}
tf-idf / +svd / k-nn	Directed	60251	3274	13.4	155	27	7.34	3.42	0.297	0.008
	Undirected	55148	4702	23.5	171	7	3.69	2.96	0.245	0.005
tf-idf / +svd / cs	Directed	59613	3358	12.8	138	24	7.62	3.48	0.282	0.008
	Undirected	54758	4702	23.3	185	7	3.67	2.96	0.237	0.005
ppmi / +svd / k-nn	Directed	60250	4414	12.9	97	20	5.61	3.57	0.273	0.006
	Undirected	47740	4702	20.3	102	7	3.83	3.09	0.259	0.005
ppmi / +svd / cs	Directed	59613	4474	12.7	106	19	5.77	3.60	0.251	0.006
	Undirected	48504	4702	20.6	108	8	3.76	3.07	0.242	0.005

Note. m = number of edges; n_{CC} = number of nodes of the largest (strongly) connected component; $\langle k \rangle$ = average node degree; k_{max} = maximum node degree; D = diameter of the network; L = average shortest path length; L_{random} = average shortest path length of the random network with the same size and density; C = clustering coefficient; C_{random} = clustering coefficient of the random network with the same size and density.
