# Supplementary material for: A Complex Network Approach to Distributional Semantic Models
Source: PLoS One. 2015 Aug 21;10(8):e0136277. doi: 10.1371/journal.pone.0136277 (PMC4546414; doi:10.1371/journal.pone.0136277)
Supplement: S2 Table — (PDF) [file pone.0136277.s002.pdf]

Table S2. Detailed results of statistical tests of power-law behavior for the degree distributions of all 24 DSM networks.

weighting / smoothing / neighborhood method	Goodness-of-fit test for the power law k_{min}	α	D_{KS}	p	Average log-likelihood by 10-fold cross-validation: power law	truncated power law	exponential	Result code
Word-document matrix								
unweighted / -svd / k-nn	21	2.79	0.025	.26	-246.68	-237.13	-267.79	+T
unweighted / -svd / cs	13	2.38	0.020	.45	-323.23	-323.24	-362.66	+PT
tf-idf / -svd / k-nn	18	2.66	0.023	.29	-303.36	-280.35	-333.79	+T
tf-idf / -svd / cs	12	2.34	0.019	.50	-325.22	-325.22	-369.19	+PT
ppmi / -svd / k-nn	23	2.69	0.017	.92	-217.66	-217.68	-230.84	+PT
ppmi / -svd / cs	14	2.34	0.023	.28	-305.76	-305.75	-337.10	+PT
unweighted / +svd / k-nn	47	5.76	0.030	.94	-33.50	-33.52	-33.61	+PT
unweighted / +svd / cs	44	4.22	0.040	.46	-63.03	-62.95	-63.15	+PT
tf-idf / +svd / k-nn	36	5.68	0.028	.80	-44.41	-44.85	-45.66	+P
tf-idf / +svd / cs	40	4.57	0.035	.65	-61.18	-61.57	-62.10	+P
ppmi / +svd / k-nn	43	7.71	0.046	.47	-22.48	-22.49	-22.53	+PTE
ppmi / +svd / cs	31	4.41	0.032	.39	-105.53	-105.20	-105.33	+T
Word-word matrix								
unweighted / -svd / k-nn	22	2.35	0.029	.24	-192.05	-191.79	-199.68	+T
unweighted / -svd / cs	18	2.28	0.031	.11	-207.86	-207.55	-217.56	+T
tf-idf / -svd / k-nn	39	3.47	0.091	.06	-24.92	-24.74	-24.76	−TE
tf-idf / -svd / cs	9	2.12	0.097	.00	-69.66	-68.25	-68.62	−T
ppmi / -svd / k-nn	33	3.52	0.043	.02	-153.85	-153.35	-153.95	−T
ppmi / -svd / cs	38	3.75	0.034	.46	-100.56	-100.49	-101.37	+PT
unweighted / +svd / k-nn	45	3.20	0.030	.70	-83.82	-83.72	-84.57	+T
unweighted / +svd / cs	43	3.12	0.043	.15	-100.89	-100.71	-101.76	+T
tf-idf / +svd / k-nn	40	4.45	0.027	.88	-70.19	-70.14	-70.51	+PT
tf-idf / +svd / cs	39	4.51	0.031	.74	-62.91	-62.88	-63.25	+PT
ppmi / +svd / k-nn	31	5.30	0.027	.49	-110.76	-110.60	-110.78	+T
ppmi / +svd / cs	31	5.21	0.027	.60	-95.57	-95.52	-95.86	+PT

Note. Codes in the last column denote the results of statistical tests. A detailed description of the coding scheme is given in the main body of the paper and Table 3. Red and green cells denote that the in-degree distribution follows the pure power law and the truncated power law, respectively. k_{min} = lower bound to the power-law behavior; α = power-law exponent; D_{KS} = Kolmogorov–Smirnov distance between the data and the theoretical power-law fit.
