# Supplementary material for: A Complex Network Approach to Distributional Semantic Models
Source: PLoS One. 2015 Aug 21;10(8):e0136277. doi: 10.1371/journal.pone.0136277 (PMC4546414; doi:10.1371/journal.pone.0136277)
Supplement: S1 Fig — (a) in-degree distributions of DSM networks generated from the word-document matrix, (b) in-degree distributions of DSM networks generated from the word-word matrix, (c) cumulative in-degree distributions of DSM networks generated from the word-document matrix, (d) cumulative in-degree distributions of DSM networks generated from the word-word matrix. (PDF) [file pone.0136277.s003.pdf]

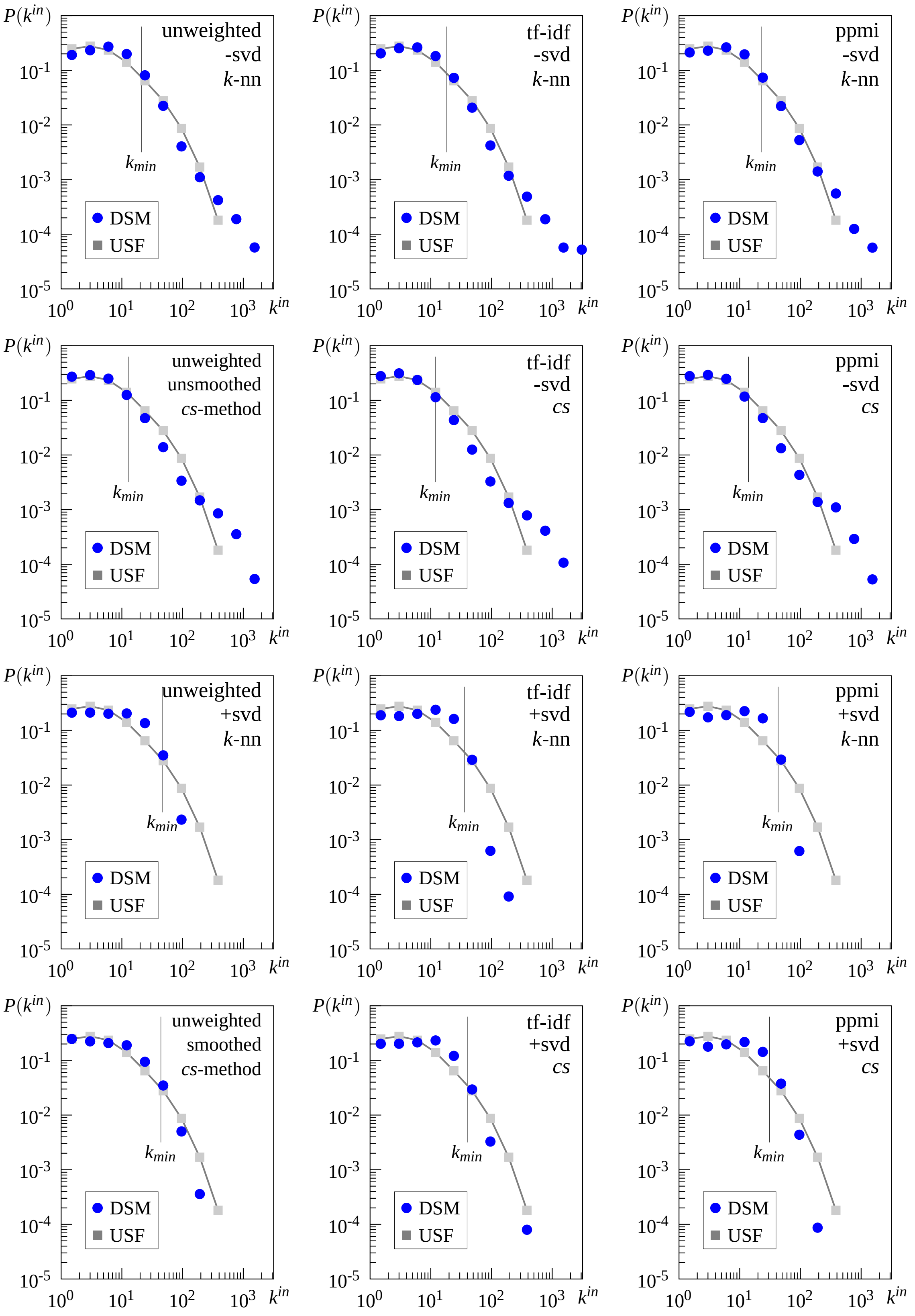

(a) In-degree distributions of DSM networks generated from the word-document matrix.

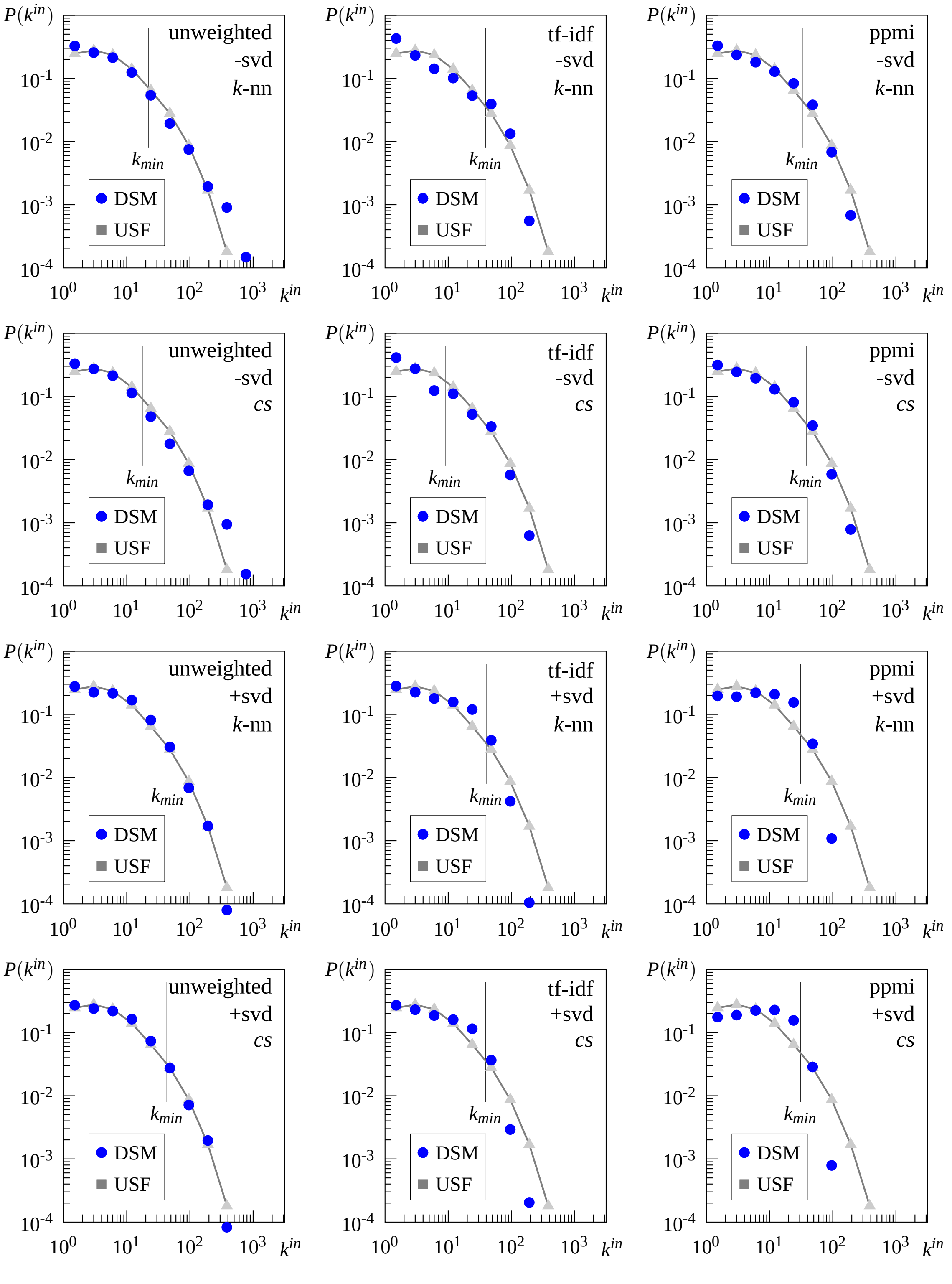

(b) In-degree distributions of DSM networks generated from the word-word matrix.

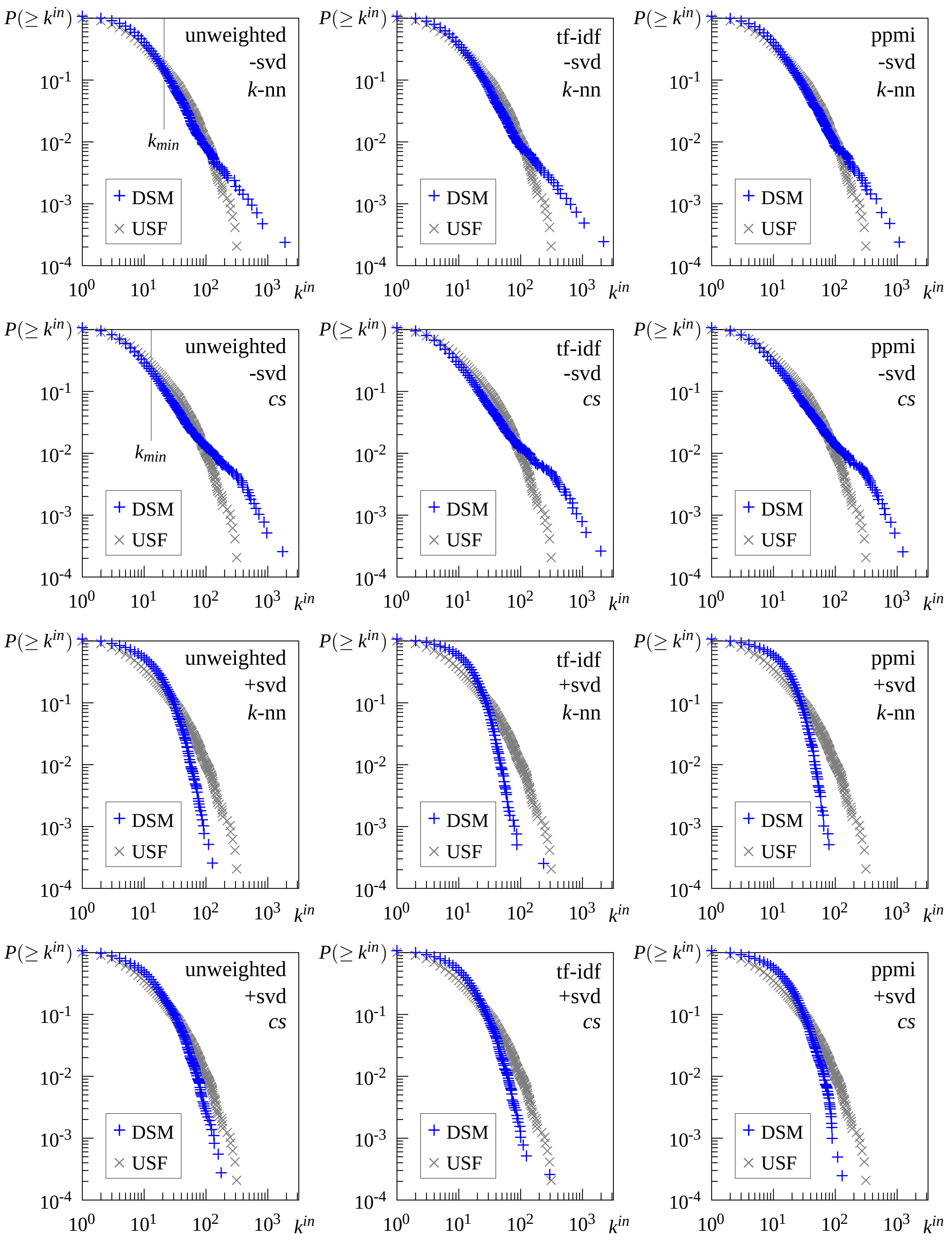

(c) Cumulative in-degree distributions of DSM networks generated from the word-document matrix.

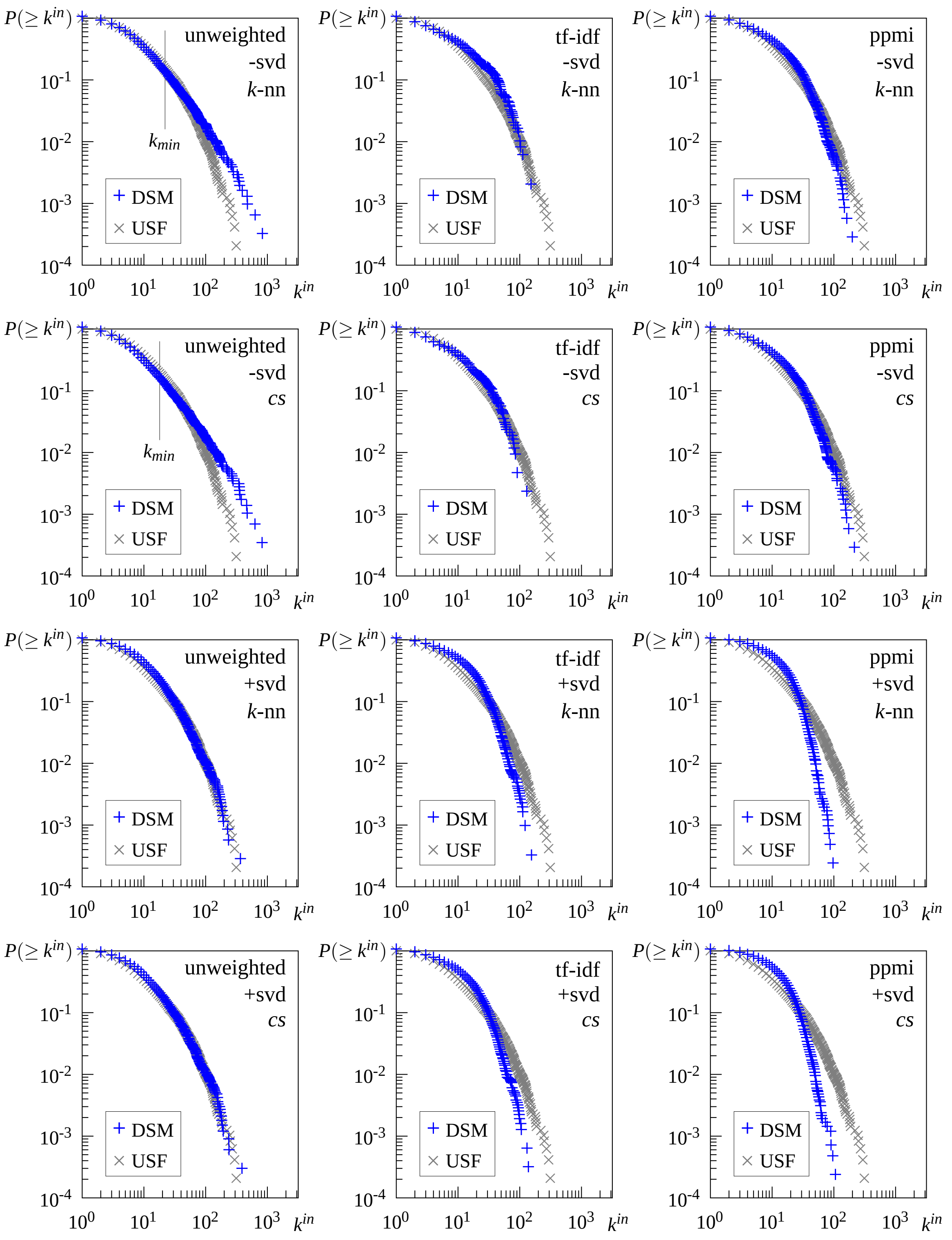

(d) Cumulative in-degree distributions of DSM networks generated from the word-word matrix.
