# Supplementary material for: A Complex Network Approach to Distributional Semantic Models
Source: PLoS One. 2015 Aug 21;10(8):e0136277. doi: 10.1371/journal.pone.0136277 (PMC4546414; doi:10.1371/journal.pone.0136277)
Supplement: S2 Fig — (a) local clustering coefficient as a function of the node degree for DSM networks generated from the word-document matrix, and (b) local clustering coefficient as a function of the node degree for DSM networks generated from the word-word matrix. Red plots denote the local clustering coefficient of an individual node, the blue line connects the average of the local clustering coefficient with the same degree, and the dashed line denotes the clustering coefficient C. (PDF) [file pone.0136277.s004.pdf]

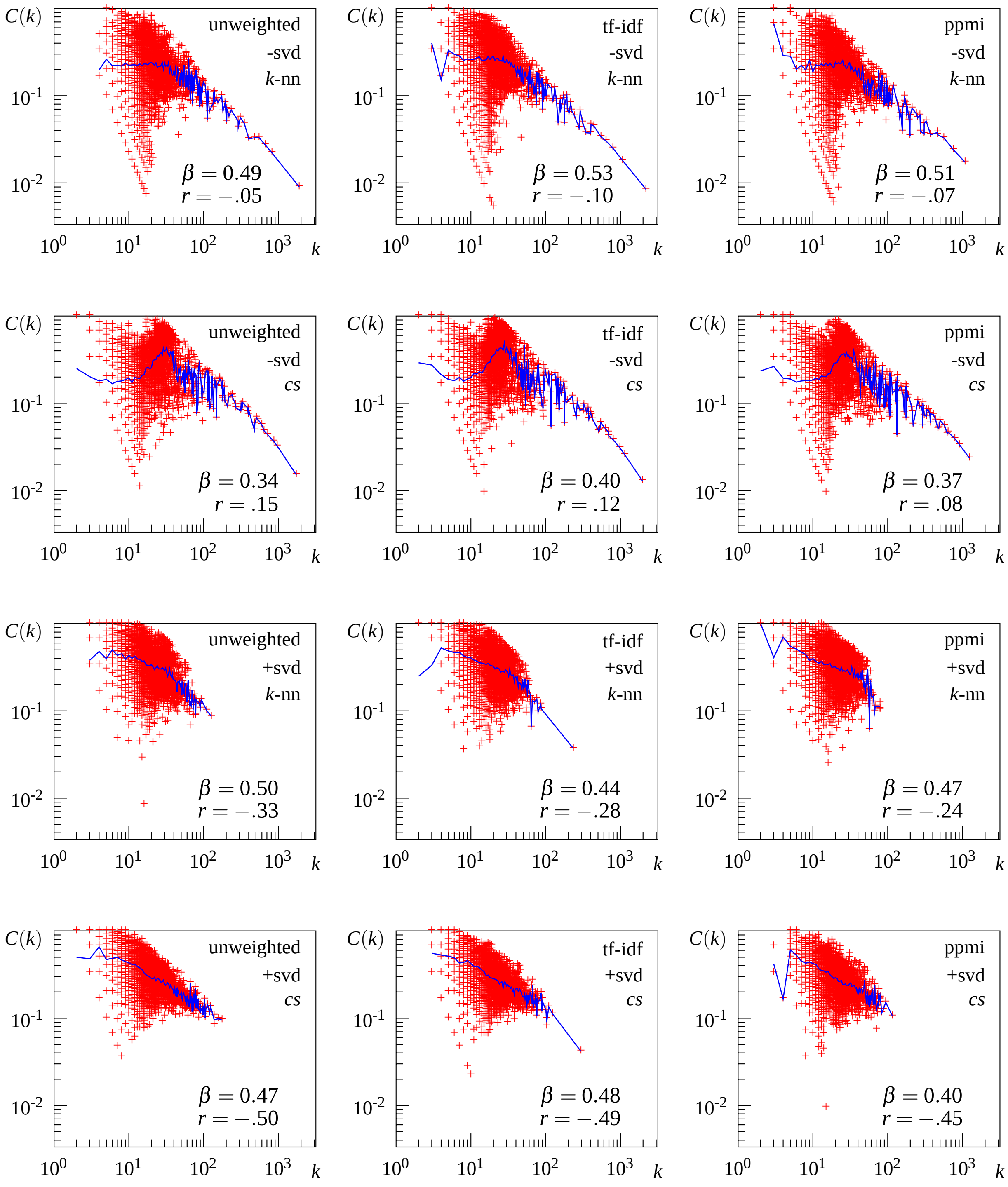

(a) Local clustering coefficient as a function of the node degree for DSM networks generated from the word-document matrix.

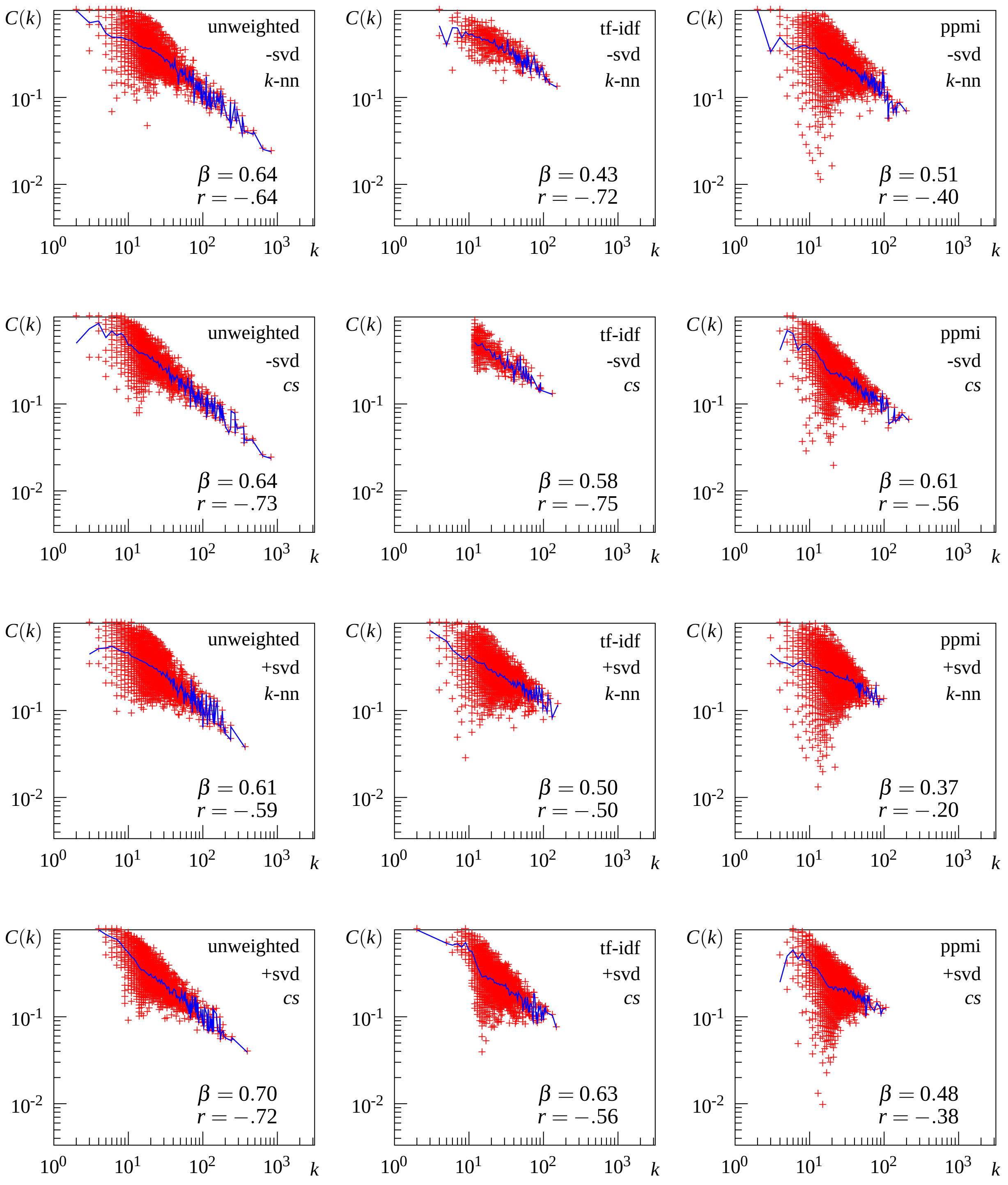

(b) Local clustering coefficient as a function of the node degree for DSM networks generated from the word-word matrix.
